# Experimental Research on Concrete Beams Reinforced with High Ductility Steel Bars and Strengthened with a Reactive Powder Concrete Layer in the Compression Zone

**DOI:** 10.3390/ma13184173

**Published:** 2020-09-19

**Authors:** Zbigniew Perkowski, Mariusz Czabak, Stefania Grzeszczyk, Daniel Frączek, Karolina Tatara, Aneta Matuszek-Chmurowska, Krystian Jurowski, Bronisław Jędraszak

**Affiliations:** 1 Department of Physics of Materials, Faculty of Civil Engineering and Architecture, Opole University of Technology, Katowicka 48, 45-061 Opole, Poland; d.fraczek@po.edu.pl (D.F.); k.tatara@po.edu.pl (K.T.); 2 Department of Building Materials Engineering, Faculty of Civil Engineering and Architecture, Opole University of Technology, Katowicka 48, 45-061 Opole, Poland; s.grzeszczyk@po.edu.pl (S.G.); a.matuszek-chmurowska@po.edu.pl (A.M.-C.); k.jurowski@po.edu.pl (K.J.); 3 Department of Mechanics and Structural Engineering—Structural Engineering Laboratory, Faculty of Civil Engineering and Architecture, Opole University of Technology, Katowicka 48, 45-061 Opole, Poland; b.jedraszak@po.edu.pl

**Keywords:** ultra-high performance fibre reinforced concrete, reactive powder concrete, composite beam, destructive tests, displacement measurement, strain measurement, curvature measurement, ultrasonic tests, photogrammetric analysis, cracks, reinforcing steel of high ductility

## Abstract

The article describes four-point bending tests of three reinforced concrete beams with identical cross-sections, spans, and high-ductility steel reinforcement systems. Two beams were strengthened in the compressed section with a thin layer of reactive powder concrete (RPC) bonded with evenly spaced stirrups. Their remaining sections, and the third reference beam, were made of ordinary concrete. Measurements of their deflections, strains and axis curvature; ultrasonic tests; and a photogrammetric analysis of the beams are the main results of the study. For one of the beams with the RPC, the load was increased in one stage. For the two remaining beams, the load was applied in four stages, increasing the maximum load from stage to stage in order to allow the analysis of the damage evolution before reaching the bending resistance. The most important effect observed was the stable behaviour of the strengthened beams in the post-critical state, as opposed to the reference beam, which had about two to three times less energy-absorbing capacity in this range. Moreover, thanks to the use of the RPC layer, the process of concrete cover delamination in the compression zone was significantly reduced, the high ductility of the rebars was fully utilized during the formation of plastic hinges, and the bending capacity was increased by approximately 12%.

## 1. Introduction

Reactive powder concrete (RPC) is one of the most advanced achievements in concrete technology. The composition and properties of RPC were first presented in the world literature by Richard and Cheyrezy in 1995 [1], although historically, the research leading to this achievement of material engineering was initiated already in the early 1970s [2]. Depending on the strength obtained, RPC can be classified as very high performance concrete (VHPC: compressive strength 100–150 MPa [3]) or ultra-high performance concrete (UHPC: compressive strength above 150 MPa [3]), and using steel fibres, it can be classified as ultra-high performance fibre-reinforced concrete (UHPFRC [2]). The compressive strength of RPC with steel fibres is usually around 200 MPa, but with proper heat and pressure treatment with quartz aggregate, it can achieve up to around 500 MPa, and even up to 800 MPa with steel aggregate. Other mechanical parameters are also beneficial. For example, for RPC of strength class 200, its tensile strength is on average 30–60 MPa, its Young’s modulus is 50–60 GPa, its fracture energy is 20–40 kJ/m^2^, and its ultimate tensile strain is 0.005–0.007 [1]. To this day, a number of subsequent studies of various varieties of UHPC and UHPFRC have been carried out, whose formulas were based on the RPC concept according to [1], primarily focusing on their strength characteristics (e.g., [3,4,5,6,7,8,9,10,11,12,13,14,15,16,17,18,19,20]), as well as other important properties (e.g., deformability [3,5,6,8,11,12,14,16,17,18,19,20], porosity [16,17], freeze-thaw durability [14,18], workability [9,13,18], resistance to projectile penetration [21], parameters of fracture mechanics [7,12,16], creep [10,22], shrinkage [6,22], adhesion to ordinary concrete [11,14,23] and to reinforcing steel [14,24], resistance to abrasion [14], evaluation of microstructure [15,16]), and confirming at the same time the very favorable characteristics of this type of concrete from the point of view of construction engineering.

On the basis of the quoted literature, the following general facts concerning the manufacturing technology and properties of RPC can be established. Its high strength can be achieved with the right choice of ingredients. This concrete requires a relatively high content of cement (usually 800 to 1000 kg/m^3^), silica fume (20–30% by the weight of the cement) and quartz powder. RPC does not contain large aggregates, and the average size of its aggregate particles is about 500 μm. The use of dispersed reinforcement in the form of steel fibres (1.5 to 3.0% by volume) and a low w/c ratio (typically around 0.2) is also required, which is achieved by using the latest generation of superplasticisers based on carboxylates. The replacement of large aggregate with sand and the use of quartz powder and silica fume in RPC allow us to eliminate the transition layer between the sand particles and the cement paste, and to obtain a homogeneous and tight microstructure in this material. On the other hand, the low w/c ratio results in the presence of a significant amount of unreacted cement particles in the concrete, which—in the case of forced cracks in this composite—may further hydrate, favorably closing the free transfer for liquids and gases from outside into the concrete, especially migration of water and aggressive solutions. A tight and homogeneous microstructure prevents the penetration of corrosive agents and allows us to obtain material a with very low porosity. As a consequence, this leads to a significant minimisation of the evolution of mechanical, thermal, or chemical microcracking and thus to an increase in the durability of the concrete. Since the production of RPC is associated with high consumption of Portland cement (and thus also with a relatively large carbon footprint and costs), there are also examples in the research problem literature of so-called green RPC, with a reduced amount of this binder in favour of finely ground slag [6,7] and fly ashes [7].

As mentioned above, RPC is characterised by very high compressive strength and relatively high tensile strength and stiffness. For this reason, the dimensions of the cross-sections of the structural elements and the weight can be significantly reduced, or the traditional reinforcement can be reduced, or even completely reduced in some cases. As a consequence, it also allows us to create structural elements of architecturally interesting shapes. An example is the footbridge in Sherbrooke (Canada), erected in 1997, where RPC was used for the first time as a building structure [25], or the precast façade elements of Stade Jean Bouin in Paris [26]. Other examples of interesting RPC applications that can be mentioned are deep foundations [27], coastal engineering [27], and composite bridge structures in which the joints between the precast members are filled with UHPC [28]. A number of already successful UHPC and UHPFRC applications in practice, and their high strength and durability, continue to lead many researchers to undertake research proposing various innovative structural solutions using such materials, including their use for strengthening existing structures. In this respect, concrete beam structures [5,6,10,11,12,13,19,20,29,30,31,32,33], as well as columns [8,34,35] and slabs [9,36] have mainly been examined. In addition, analytical [12,29,33,36] and numerical [6,11,19,20,30,32] models of elements containing UHPC or UHPFRC have been verified or validated, the properties of this material have been tested for use in repairs [14,37], and the possibilities of the modern monitoring of the structural elements which contain it have been tested [12]. It is worth mentioning here that there are also papers in which UHPC or UHPFRC is used as a component of composite beams where it is combined with glued-laminated timber [32,33] or fibre-reinforced polymer [31]. However, the largest number of studies in this area concern reinforced concrete composite beams consisting of ordinary concrete (OC) and RPC in different configurations of their layers. For example, the RPC layers in the beam have been added on its tension face [11,12,13,19,29], compression face [19], in the form of a ‘U’ [11], or only on the lateral sides of the cross-section [11]. They have contained additional longitudinal reinforcement [13,19] or not [11,12,13,19]; they have been fixed with epoxy resin [11,12,13], anchors [13], or the contact consists mainly in the adhesion forces between OC and RPC [11,19]. On the other hand, in [9], one-way-reinforced OC slabs with the bottom layer of RPC with or without additional longitudinal reinforcement were tested, and in [36], two-way-reinforced OC slabs with a top two-way-reinforced layer of RPC. In [9], in order to join the OC and RPC layers, several stirrups penetrating both layers in the shear zones were used in part of the tested slabs, basically depending on the adhesion forces between the concrete layers, and in [36], the contact of the layers was strengthened by means of evenly distributed studs. A broad overview of the above-mentioned studies can be found in paper [38]. Most often, the results of the above-mentioned works show the beneficial effects of the increased load-bearing capacity of the tested structural elements and/or increased energy absorption as a result of using additional RPC. According to the authors of this paper, it can be concluded on this basis that the creation of concrete composite structures with RPC is a particularly beneficial way to use it in practice for both the manufacture of precast elements and the strengthening of the existing ones. Creating OC–RPC composite structures instead of structures made only of RPC is justified in view of the need to reduce costs and carbon footprint due to increased binder consumption and the higher technological requirements for RPC production (e.g., according to [39], the cost of RPC with a strength of 90–140 MPa compared to OC with a compressive strength of 30–60 MPa is approx. 3–5 times higher). The most obvious solution in this case is therefore to reduce the consumption of this material and replace it in places where its presence is not required for structural reasons by another, cheaper type of concrete. However, it is important that this should be done prudently and skillfully, as can be seen in the context of some experimental research. For example, in the aforementioned publication [9], particularly interesting results in this respect concerned the series of slabs (denoted in [9] as the RE series) bent at three points with a cross-section of 300 mm × 100 mm and length of 1600 mm, with the same longitudinal reinforcement system (tension reinforcement ratio approx. 2.5%), while the thickness of bottom strengthening RPC layers with compressive strength of about 150 MPa was differentiated (20, 32, or 50 mm). The rest of the section was comprised of OC with a compressive strength of 23 MPa. The centre of gravity of the cross-section of the longitudinal main reinforcement (made of 12 mm diameter bars spaced at 62 mm intervals) was 26 mm from the tension face of the element, so that the rebars went beyond the thinnest layer of RPC. Interestingly, the load capacities of the slabs with an RPC layer turned out to be lower than that of the reference slab made of OC only, while the second reference slab made entirely of RPC obtained the highest load-bearing capacity. The lack of a strengthening effect in the case of the double-layer slabs resulted, among other things, from the lack of a sufficiently load-bearing joint between the two layers of concrete. On the other hand, the slabs with RPC layers showed much greater deflection in the post-critical range than the reference OC slab. In addition, as the share of RPC in the slabs increased, the destruction of the components changed as a result of shear through the intermediate phases up to bending. In the first extreme case of slabs without RPC, diagonal and perpendicular cracks went through the entire section and were visible in many places on the underside of the slab before their load-bearing capacity was exhausted. In the case of strengthening, the propagation of almost all of the cracks formed in the OC stopped at the boundary with the RPC layer, which, in the case of the thinnest RPC layer without reinforcing bars, even led to its debonding. Eventually, when the load resistance of the RPC-strengthened slabs was reached, there was only one main perpendicular crack formed through the whole height of the cross-section in the middle section of the span. The process of destroying the strengthened slabs was therefore not clearly signaled by surface cracking in the view from below. In turn, in the study [19], the four-point bending of a series of beams of a cross-section of 250 mm × 400 mm and a length of 3000 mm with identical reinforcement arrangements (tension reinforcement ratio approx. 0.45%) was carried out. Their strengthening was varied and applied with 20, 40, or 60 mm thick top or bottom layers made of RPC with a compressive strength of about 156 MPa (the beam series denoted in [19] as BU and BL, respectively). The rest of their cross-section was comprised of OC with a compressive strength of about 30 MPa. The center of gravity of the cross-section of the longitudinal bottom reinforcement (made of 2 bars with a diameter of 16 mm) was located 40 mm from the tension face of the element. The longitudinal top reinforcement was located analogously from the compression face. It has been shown, as in [9], that most of the cracks developed in the OC stopped at the boundary with the RPC layer, leading to the partial delamination of the joint. This phenomenon in the case of strengthening with the lower layer of RPC means again that there is no clear signaling processes leading to the exhaustion of the load-bearing capacity in the underside view; all the more so as the bottom-strengthened beams showed about 2–3 times less deflection at the moment of failure than the top-strengthened beams, and all of them showed less ultimate deflection than the reference beam made entirely of OC. On the other hand, when using an RPC layer, the beams showed a higher load-bearing capacity in the range up to about 30% compared to the reference beam, except for the beam with the thinnest RPC layer from the bottom, which showed no increase in load-bearing capacity at all.

The discussed research examples show how important it is to skilfully combine two layers of concrete with extremely different properties in a structural element. For the authors of this paper, it was, among other things, a contribution to performing their own research in this area. It was focused on the search for a way to combine OC and RPC layers, and their arrangement together with reinforcing bars in the beam cross-section, which will enable the economical and effective use of RPC, so that the process of overloading the element is at the same time clearly visually signaled by cracks on the tension face of the element. Of course, the latter is possible in a situation where the longitudinal tension reinforcement is yielded before the concrete is crushed in the compressed part. It is also the classic preferred way of destroying beam structures for safety reasons in reinforced concrete mechanics if they are overloaded (e.g., [40]). In addition, with today’s typical use of high-ductility steel (class C according to EN 1992-1-1 [41]), such a destruction mechanism will naturally allow for high energy absorption in the post-critical state, and the full utilisation of the potential of the reinforcing steel while using an RPC with very high compressive strength in the compressed beam’s part. Therefore, on the basis of the literature review (e.g., [9,19]), the use of simply supported OC beam strengthened with an RPC layer on its bottom tension face was abandoned, and it was applied only in the top layer. In addition, due to the possibility that a relatively thin layer of RPC may be delaminated as a result of the development of cracking and the premature destruction of the compression layer, a stirrup connection was applied along the entire length of the beam.

Another aspect that was considered in the work was the optimal choice of the longitudinal tension reinforcement ratio. In the literature available to the authors, no further analyses on this subject have been conducted so far in the case of laboratory tests of OC beams with RPC strengthening. For this reason, before determining the final layout of the longitudinal tension rebars, a simplified economic and strength analysis was first carried out, based on which a reinforcement ratio of 1.7% was applied. The results obtained from the four-point bending of the strengthened beams were compared with a reference beam made entirely of OC. The research was also supported by the photogrammetric analysis of the appearing cracks, where the original method of image processing was proposed and used in this respect. An additional new element compared to the studies available in the literature is that one of the strengthened beams and the reference beam were loaded and unloaded in stages while increasing the maximum load in subsequent stages. This procedure was introduced in order to enable, during the full unloading between the stages, the supplementary ultrasonic testing of the concrete’s condition in the middle section of the beams with a constant bending moment. As a result, it enabled a more complete assessment of the development of brittle concrete damage and its comparison with information on the condition of the beams, which results from parallel measurements of strains and deflections. In this respect, the experience gained by the authors, which was described in [42,43], was followed in order to obtain knowledge about the change of the properties of the tested elements before their load resistance is reached in the context of possible diagnostic applications.

To make the reading of the article more convenient, Appendix A—a list of the most important symbols used in this work for mathematical and physical quantities—is provided.

## 2. Materials

This chapter describes the materials that were used in the construction of reinforced concrete beams, i.e., OC from a local concrete plant, high-ductility reinforcing steel, and the RPC. It contains introductory information about their basic parameters, as it was the starting point for the authors to establish the most important assumptions about the geometry and testing method of the beams, as presented in the next chapter. Moreover, because the RPC was prepared entirely in the Department of Building Materials Engineering of Opole University of Technology, the main attention was devoted to the discussion of its recipe and the tests of the selected properties, which showed its high suitability for structural applications.

### 2.1. RPC

For the preparation of the RPC mixture, the following were used: Portland cement CEM I 52.5 R from Rejowiec Cement Plant (Poland) with a specific surface area of 410 m^2^/kg, silica fume (0/45 μm) from Łaziska Steelwork (Poland); quartz powder (0/0.2 mm) and quartz sand (0/0.5 mm) from an aggregate mine in Osiecznica (Poland); a BASF superplasticiser based on polycarboxylates (2.5% by weight of cement); and steel fibres with the trade name WHS-12/0.2, a length of 12 mm, and a diameter of 0.2 mm. The chemical composition of the cement, quartz powder, and quartz sand is presented in Table 1. The optimisation of the composition of the concrete mixture, in order to increase the packing of its particles, was based on the Funk–Dinger curve [44]. The composition of the RPC mixture is presented in Table 2.

The RPC consistency tests were carried out in accordance with EN 1015-3 [45], based on measurement of the mixture’s flow diameter. The compressive and bending strength tests were carried out for six samples, in accordance with EN 1015-11 [46], with the use of a Controls MCC8 strength machine. Prisms of 40 mm × 40 mm × 160 mm were made for testing. After 24 h of storing the samples in molds under normal conditions (temperature 20 ± 2 °C, air humidity 60 ± 5%), the samples were demoulded and immersed in water at 20 ± 2 °C. The samples were taken out of the water and subjected to strength tests after 2, 7, and 28 days. The test of the freeze–thaw durability of the concrete in the presence of de-icing salt (3% NaCl) was carried out for 4 samples according to technical specification CEN/TS 12390-9 [47]. In turn, the tests of the RPC microstructure were carried out with the NOVA NANO SEM 200 scanning electron microscope. A layer of gold was sprayed onto the samples. X-ray microanalyses were performed in selected micro-areas. The porosity of the concrete samples was tested for 3 samples with the PoreMaster 60 mercury porosimeter in the pressure range from 1 to 400 MPa, which allowed us to determine the pore size volumetric share and the pore size distribution curve in the range from 0.0035 μm to 1000 μm.

The results of the concrete mix consistency tests using the flow table test have shown that it maintains its liquidity allowing it to be laid in one hour. The diameter of the flow after one hour was 250 mm. 

The results of compression and flexural strength testing of the RPC samples are shown in Table 3 where it can be seen that the RPC reaches a compressive strength of about 146 MPa after only 2 days, while after 28 days the strength reaches about 200 MPa. The concrete also achieves a high flexural strength, which after 28 days is about 50 MPa. 

The results of the resistance tests of the RPC to frost and de-icing agents showed very high resistance to these factors. The weight of scaling of the concrete sample after 56 freezing–thawing cycles was only 0.0007 kg/m^2^. This allows the freeze–thaw durability of the concrete to be assessed as ‘very good’ according to the criteria adopted in technical specification CEN/TS 12390-9 [47].

**Figure 1:**
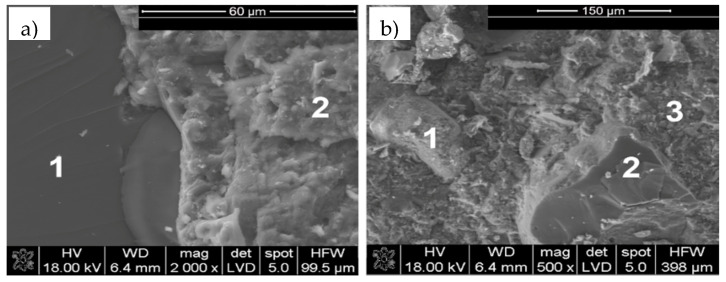
Microstructure of the RPC after 28 days of maturation with a visible C–S–H phase closely adhered to the quartz particles. (**a**) C–S–H phase (point 2) and quartz particle (point 1). (**b**) C–S–H phase (points 1 and 3) and quartz particle (point 2).

**Figure 2:**
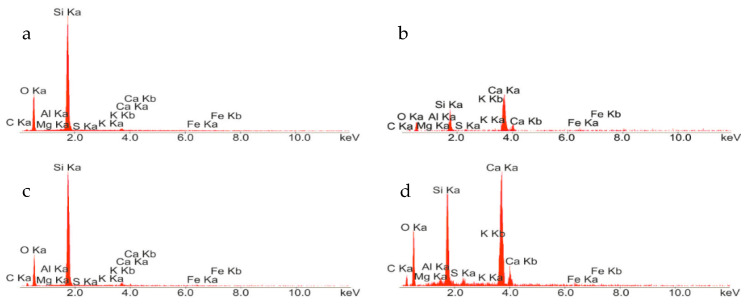
X-ray microanalysis: (**a**) in point 1 of Figure 1a: a quartz particle, (**b**) in point 2 of Figure 1a: C–S–H phase, (**c**) in point 2 of Figure 1b: quartz particle, (**d**) in point 1 of Figure 1b: C–S–H phase.

The results of the microstructure tests of the concrete samples—as obtained by scanning microscopy and X-ray microanalysis after 28 days of maturation—are presented in Figure 1 and Figure 2, respectively. Observations of the RPC microstructure showed primarily the presence of the compacted C–S–H phase (point 2 in Figure 1a, points 1 and 3 in Figure 1b, X-ray analysis in Figure 2b,d), which is responsible for the concrete’s high strength and durability. It is formed as a result of the reaction of calcium ions with silica fume during the cement’s hydration, and it adheres very closely to quartz particles (point 1 in Figure 1a, point 2 in Figure 1b, X-ray microanalysis in Figure 2a,c).

**Figure 3:**
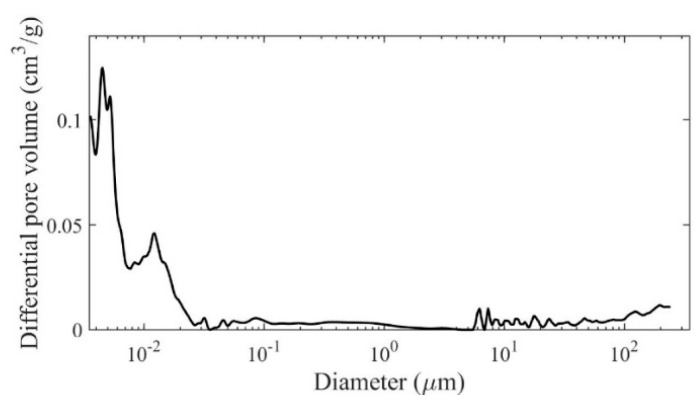
Differential curve of pore volume distribution in the function of the pore diameter in the RPC after 28 days of maturation.

The results of the total porosity and size pore volumetric share in the concrete samples, as determined by mercury porosimetry after 28 days, are presented in Figure 3 and Table 4. As shown in Table 4, the total volume of the pores in the RPC concrete sample after 28 days is small, and amounts to 4.4%. What attracts interest is the definite advantage in the RPC’s microstructure of mesopores below 20 nm in diameter, which occupy 77.1% of the total pore volume. The number of larger pores with diameters between 20 and 20,000 nm, which occupy 14.1% of the total pore volume, is much smaller. The decrease in the proportion of larger pores, with a significant increase in the proportion of mesopores, favourably influences the strength and durability of RPC.

### 2.2. Ordinary Concrete

The OC was ordered from a concrete plant located in Opole (Poland). The concrete mixture had a consistency of F3, as determined by the flow table test according to EN 12350-5 [48]. According to the information provided by the manufacturer, the concrete mix formula used natural aggregate with a maximum particle size of 16 mm, from the aggregate mine nearby Opole (Poland), and blast-furnace cement CEM III A 42.5N HSR according to EN 197-1 [49] from Odra Cement Plant in Opole (Poland), and the water–cement ratio was 0.6. The mechanical properties of the concrete, i.e., the compressive strength and secant modulus of elasticity were determined with the use of a Controls MCC8 strength machine according to EN 12390-3 [50] and EN 12390-13 [51], respectively. For this purpose, 3 samples for each kind of tests were taken from the same batch of concrete that was used to prepare the beams, stored under the same conditions, and tested on the same day on which the beam bending test was performed. The average compressive strength of the concrete was determined on cubic samples with a side of 150 mm was 65 MPa, which allowed us to assign it a strength class of C45/55 on the test day, according to EN 1992-1-1 [41]. The stabilised modulus of elasticity was determined on cylindrical specimens with a diameter of 150 mm and a height of 300 mm, and its average value was 30.4 GPa. 

### 2.3. Reinforcing Steel

Bars made of the B500SP steel were used for the reinforcement of the beams. The steel was compliant with the standard [52], and was manufactured in Poland with the Epstal certificate. As declared by the manufacturer, it has the following parameters: a minimum yield point of 500 MPa, a ratio of tensile strength to yield point in the range 1.15–1.35, and a minimum normal strain at the maximum load of 0.08, which allows us to classify it as steel with the highest ductility class C, according to EN 1992-1-1 [41]. The main longitudinal reinforcement in the tension zone of the beams was made of bars with a diameter of 20 mm, the longitudinal assembly reinforcement in the compression zone of beams was made of bars with a diameter of 12 mm, and the shear reinforcement was made of two-legged stirrups with a diameter of 8 mm. Further considerations also assume that the Young’s modulus of the steel is 200 GPa, according to [41].

## 3. Preparation of the Beams

### 3.1. Optimum Longitudinal Reinforcement Ratio and Ultimate Limit State Model in the Beam with RPC Strengthening in the Compression Zone

**Figure 4:**
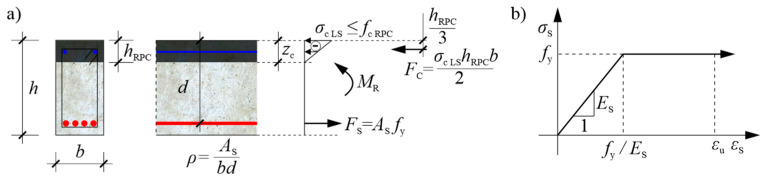
(**a**) Distribution of stresses and their resultants for the assumed ultimate limit state under bending in the reinforced concrete beam strengthened with the RPC layer in the compression zone ($z_{c}$ is the position of neutral axis as its distance [m] from the beam’s compression face equal the height of RPC layer in the limit state). (**b**) Assumed stress–strain relation in the reinforcing steel (based on [41]).

It was decided in the study that the tested beams should have a tension reinforcement ratio for bending as close as possible to the optimal one, and the strengthening of the RPC beams should be located due to the overload working conditions in the compression zone only (see Section 1). Typically, the optimum reinforcement ratio of the beam is determined taking into account costs, while maintaining the bending moment resistance condition (e.g., [53]), which for the purpose of this paper was adopted on the basis of the documentation of cracks on the loaded beams included in [19]. The authors assumed, in this respect, that the ultimate limit state under bending occurs when a neutral axis reaches the boundary of the OC and RPC layers, i.e., when the cracks in the tensioned OC approach the layer with the RPC, and this layer still remains entirely in the compression zone. Then, the tensile normal stress in the cross-section through the cracks falls entirely to zero in the OC, and the tensile stress just below the layers’ boundary in the OC are negligible, and can be ignored. In contrast, given the significant compressive strength of the RPC, the tensile stress in the longitudinal main steel reinforcement reaches its yield point. Since, typically, the stress–strain relation in compressed RPC is approximately linear until the compressive strength is reached, the plastic flow plateau is very limited or absent, and when the strength limit is reached with a further increase in strain, the stress falls rapidly (e.g., [16,19]), the authors have adopted a linearly-variable relation between compressive stress and normal strain in the RPC, while stress cannot reach the compressive strength value in the extremely compressed beam fibres. For the sake of simplicity, maintaining the planarity of the beam’s cross-sections was also adopted, in accordance with the Bernoulli–Euler beam theory. The idealisation of the distribution of normal stresses and their resultants in the cross-section of the singly-reinforced beam is shown in Figure 4a. Only the case of a rectangular cross-section with reinforcing bars arranged in one horizontal row was considered due to the scope of experimental research, and the strain hardening of steel was omitted for the sake of simplicity, assuming a horizontal section in the stress–strain relationship after reaching the yield point (Figure 4b). These assumptions make it possible to clearly estimate the range of compressive stresses on the basis of the equilibrium condition of the horizontal internal forces (stress resultants) in the cross-section:

(1) $$F_{s}=F_{c}\to A_{s}f_{y}=-\frac{1}{2}bh_{\mathrm{RPC}}\sigma_{c\;\mathrm{LS}}\to \sigma_{c\;\mathrm{LS}\;}=-\frac{2A_{s}f_{y}}{bh_{\mathrm{RPC}}}\to \sigma_{c\;\mathrm{LS}}=-\frac{2\rho df_{y}}{h_{\mathrm{RPC}}}\;\mathrm{and}\;\left|\sigma_{c\;\mathrm{LS}}\right|\leq f_{c\;\mathrm{RPC}},$$

where:

(2) $$\rho =\frac{A_{s}}{bd}\;\mathrm{and}\;d=h-a,$$

$F_{s}$ and $F_{c}$ are the resultants [N] of normal stress in the tension steel rebars and compressed concrete, respectively; $A_{s}$ is the cross-sectional area [m^2^] of the tension rebars; b and h are the width [m] and height [m] of the beam’s cross-section, respectively; $h_{\mathrm{RPC}}$ is the RPC layer thickness [m]; d is the effective depth [m] of the beam’s cross-section; a is the distance [m] of the centre of gravity of the longitudinal main rebars’ cross-section from the tension face of the beam; ρ is the longitudinal tension reinforcement ratio [-]; $f_{y}$ is the yield point [Pa] of the reinforcing steel; $f_{c\;\mathrm{RPC}}$ is the compressive strength [Pa] of the RPC; and $\sigma_{c\;\mathrm{LS}\;}$ is the stress [Pa] in the concrete at the compression face of the beam at the moment of reaching the ultimate limit state according to Figure 4a.

It should be clearly stated that the above dependencies will be correct if the normal strain in the rebars meets the following conditions:

(3) $$\frac{f_{y}}{E_{s}}\leq \varepsilon_{s}\leq \varepsilon_{u},$$

where $E_{s}$ is the modulus of elasticity [Pa] of reinforcing steel, $\varepsilon_{u}\;$ is the normal strain [-] in the reinforcing steel at maximum load (Figure 4b), $\mathrm{and}\;\varepsilon_{s}$ is the normal strain [-] in the tension rebars. In view of the adopted assumption of the maintenance of the planarity of the cross-sections, the normal strain in the steel bars, when the limit state according to Figure 4a is reached, can be estimated on the basis of the following proportions:

(4) $$\frac{-\varepsilon_{c\;\mathrm{LS}}}{h_{\mathrm{RPC}}}=\frac{\varepsilon_{s\;\mathrm{LS}}}{d-h_{\mathrm{RPC}}}\to \varepsilon_{s\;\mathrm{LS}}=-\varepsilon_{c\;\mathrm{LS}}\frac{d-h_{\mathrm{RPC}}}{h_{\mathrm{RPC}}}\to \varepsilon_{s\;\mathrm{LS}}=-\frac{\sigma_{c\;\mathrm{LS}}}{E_{c\;\mathrm{RPC}}}\frac{d-h_{\mathrm{RPC}}}{h_{\mathrm{RPC}}},$$

where $\varepsilon_{s\;\mathrm{LS}}$ is the normal strain [-] in the steel reinforcing bars at the moment of reaching the limit state; $\varepsilon_{c\;\mathrm{LS}}$ is the normal strain [-] in the concrete at the compression face of beam at the moment of reaching the limit state; and $E_{c\;\mathrm{RPC}}$ is the modulus of the elasticity [Pa] of the RPC. In the work, $\varepsilon_{s\;\mathrm{LS}}$ in the tested beams with the RPC layers was estimated using the strain measurements presented in Chapters 4–5.

The use of Equations (1)–(4) for the design of a composite beam requires that the values of b, d, $h_{\mathrm{RPC}}$, and ρ should be initially assumed, and the material parameters $E_{s}$, $E_{c\;\mathrm{RPC}}$, $f_{y}$, $f_{c\;\mathrm{RPC}}$, and $\varepsilon_{u}$ must be known. In order to reduce the design data, a $h_{\mathrm{RPC}}/h$ ratio of, e.g., 0.1–0.2 can be reasonably assumed, however, so that the two layers of the beam are connected in such a way as to prevent delamination of the thin RPC layer. If stirrups are used for this purpose, the authors propose to use $h_{\mathrm{RPC}}$ as being minimally equal to $2\cdot a_{2}$, where $a_{2}$ is the distance [m] of the center of gravity of the longitudinal secondary rebars’ cross-section from the compression face of the beam. In turn, adopting ρ, it is advantageous to rely on economic and strength criteria. For this purpose, the following objective function describing the cost of the beam’s unit section can be minimised:

(5) $$C=C_{\mathrm{RPC}}bh_{\mathrm{RPC}}+C_{\mathrm{OC}}b\left(h-h_{\mathrm{RPC}}\right)+C_{s}A_{s}\;\mathrm{and}\;h_{\mathrm{RPC}}\geq 2a_{2},$$

where $C_{\mathrm{RPC}}$, $C_{\mathrm{OC}}$, and $C_{s}$ are the price per m^3^ of RPC, OC and reinforcing steel, respectively. At the same time, it is also required that the condition for the beam’s ultimate limit state under bending is met, which results from the sum of the bending moments in its cross-section with respect to the line of action of the resultant $F_{c}$:

(6) $$M\leq M_{R}=A_{s}f_{y}\left(d-\frac{h_{\mathrm{RPC}}}{3}\right),$$

where M is the bending moment [Nm] in the beam’s cross-section, and $M_{R}$ is the beam’s bending moment resistance [Nm] according to Figure 4a. Taking into account Equations (1) and (6), a dimensionless objective function can be formulated on the basis of (5), which will be directly proportional to function (5) for the given values of $C_{\mathrm{RPC}}$, $C_{\mathrm{OC}}$, $C_{s}$, $f_{y}$, $h_{\mathrm{RPC}}=2a_{2}$, d, a, $\mathrm{and}\;M_{R}$, and will depend on the reinforcement ratio ρ and the value of the highest compressive stress $\sigma_{c\;\mathrm{LS}}$ in the concrete at the limit state; that is:

(7) $$c\left(\rho ,\sigma_{c\;\mathrm{LS}}\right)=\frac{1}{\rho \left(1-\frac{2}{3}\frac{\rho f_{y}}{\left|\sigma_{c\;\mathrm{LS}}\right|}\right)}\;\left(\rho \frac{C_{s}}{C_{\mathrm{OC}}}+\frac{h_{\mathrm{RPC}}}{d}\left(\frac{C_{\mathrm{RPC}}}{C_{\mathrm{OC}}}-1\right)+\frac{a}{d}+1\right).$$

**Figure 5:**
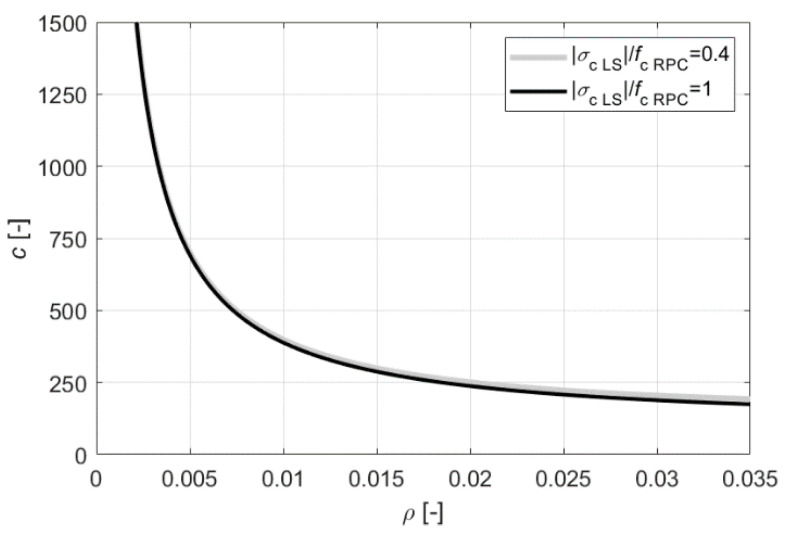
Objective function c (according to Equation (7)) depending on the reinforcement ratio ρ at different ratios of the maximum compressive stress in the RPC layer to its compressive strength $\left|\sigma_{c\;\mathrm{LS}}\right|/f_{c\;\mathrm{RPC}}$.

Figure 5 presents graphs of c for ρ in the range of up to 0.035, with the selected values of $\left|\sigma_{c\;\mathrm{LS}}\right|$ = 0.4$f_{c\;\mathrm{RPC}}$ and $\left|\sigma_{c\;\mathrm{LS}}\right|=f_{c\;\mathrm{RPC}}$, the assumed geometrical data of the reinforced beams that were prepared for the experiment (Section 3.2), the strength characteristics of the used steel and RPC, and the costs incurred by the authors in the purchasing materials. Hence, the following values are assumed: $h_{\mathrm{RPC}}/d=0.194$, a/d=0.108, $f_{y}=500$ MPa, $f_{c\;\mathrm{RPC}}=197.9$ MPa, $C_{s}/C_{\mathrm{OC}}=\;77$, $C_{\mathrm{RPC}}/C_{\mathrm{OC}}=11$. In order to obtain results close to realistic ones, the average value of $f_{c\;\mathrm{RPC}}$ and the minimum value of $f_{y}$ declared by the manufacturer were used in the calculations. It can be noted that, with reasonable data on the reinforcement ratio, the economic efficiency of the RPC-strengthened beams does not reach extremes, and decreases monotonically, but the change in cost becomes less and less significant from around ρ=0.015. The $\left|\sigma_{c\;\mathrm{LS}}\right|/f_{c\;\mathrm{RPC}}$ ratio, on the other hand, has practically no effect on the cost. Finally, for testing, it was decided to use the reinforcement ratio ρ=0.017 as the maximum at which it is still possible to use a system of longitudinal reinforcing bars with a diameter of 20 mm in one row with the required clear space between the rebars taking into account the requirements of EN 1992-1-1 [41], with a maximum aggregate size of 16 mm.

When using the formulae presented above for practical applications, it should be noted that correspondingly reduced design values should be used for $f_{y}$, $f_{c\;\mathrm{RPC}}$, and $\varepsilon_{u}$; for example, according to EN 1992-1-1 [41]. 

### 3.2. Construction of Beams

**Figure 6:**
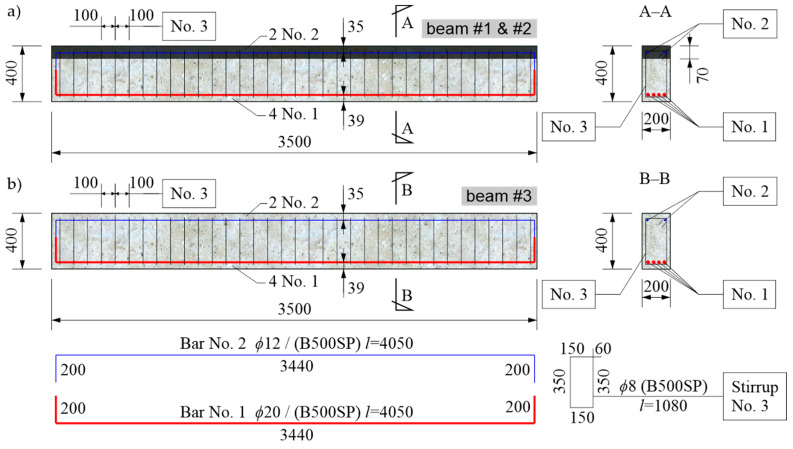
Side view, cross-section and reinforcement: (**a**) beams strengthened with the RPC layer, (**b**) reference beam (dimensions in mm).

Three 350 cm long beams with a rectangular section of 20 cm × 40 cm were prepared for the study. They were made of OC, and two of them were strengthened with a 7 cm thick RPC layer in the compression zone. It was assumed that the lower main tension reinforcement would be four bars with a diameter of 20 mm of B500SP steel. The secondary top reinforcement for the stirrup fastening was made of two bars with a diameter of 12 mm also of B500SP steel. The distance of the center of gravity of the longitudinal reinforcement section from the external surfaces of the beam was assumed to be 3.9 cm for the lower reinforcement and 3.5 cm for the upper reinforcement. The reinforcement ratio of the beams (ρ=0.017) resulted from the considerations presented in Section 3.1, while the cross-section sizes and span were selected, taking into account the equipment capabilities of the construction laboratory available to the authors. The lengths of the beams were dictated by the available space under the supporting structure of the actuators. Subsequently, their cross-sections were selected, taking into account the parameters of the 2 INSTRON PL 250 P actuators used, each of which could generate a maximum force of 250 kN. Therefore, once a symmetrical four-point bending pattern was adopted, at a distance of the force application points from the supports of 1.2 m (see Section 4.1) and leaving a reasonable margin of at least 25% of the maximum force, the anticipated real bending moment resistance of the beams could not exceed 225 kNm. As a consequence, the beams with RPC strengthening were designed so that their bending moment resistance estimated from Equation (6), as presented in Section 3.1, was 212 kNm, where the yield point for the steel was taken the minimum declared by the manufacturer ($f_{y}=500$ MPa). This moment corresponds to a load from one actuator of about 177 kN. Based on this load, the shear reinforcement for the tested elements in the form of vertical stirrups was adopted. Their number and spacing were chosen in such a way as to eliminate the effect of shear on the beams’ load-bearing capacity and, for reasons of the study, to lead to the reaching of this load level due to bending. In this respect, the formula determining the shear resistance of the elements with vertical stirrups according to EN 1992-1-1 [41] was used, and was simplified by omitting the influence of a thin RPC layer and adopting the slope of compressed concrete diagonal struts at an angle of 33.4° (averaged from the acceptable range). In order to estimate the shear resistance at a realistic level, the yield point of the stirrup steel was assumed as the minimum declared ($f_{y}=500$ MPa). Finally, two-legged stirrups with a diameter of 8 mm were adopted at a spacing of 10 cm, with an estimated shear resistance of 200 kN. The stirrup spacing every 10 cm was also maintained in the middle section of the beam. It was decided in this way to give the stirrups an additional role of joining both layers of the beam of OC and RPC, so as to prevent the thin RPC layer from delaminating due to the possible crack evolution [19] and the action of vertical tension forces at the layers’ contact in the section with a constant moment [54], and consequently to prevent its local buckling in compression. A diagram of the construction of the 2 RPC-strengthened beams is shown in Figure 6a. The third beam was made as a reference beam in relation to the first two, using the same reinforcement system, but only OC was used in this case (Figure 6b).

**Figure 7:**
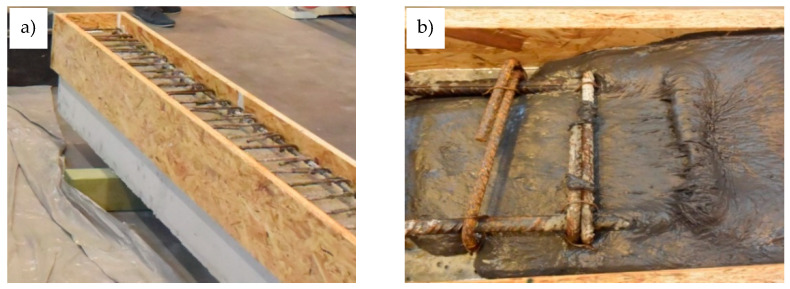
(**a**) Formwork for the RPC layer. (**b**) Pouring the RPC mixture.

In the first stage of making the elements for testing, reinforcement baskets were made and placed in the formwork prepared in the concrete plant. In the case of two beams, markers indicating the level of the OC filling were placed 7 cm from the top edge of the formwork. After the OC was poured, the beams were wet cured for 28 days, after which the formwork was dismantled, and the elements were transported to the Structural Engineering Laboratory of Opole University of Technology, where they were stored for 2 months. The temperature in the laboratory was 21 °C ± 2 °C, and the relative humidity was 60% ± 10% before the period of testing. After this period, another formwork for the upper layers of the RPC with a thickness of 7 cm (Figure 7a) was made. Then, the RPC was prepared using a laboratory mixer with a 50 L rotary bowl. Prior to pouring the RPC topping layer, the upper surface of the OC layer was moistened in order to limit the transport of water from the fresh RPC mixture into the OC layer, in order to prevent the excessive drying of the RPC mixture at the layer boundary and to ensure appropriate conditions for bonding. Due to the fact that the designed RPC mixture had a good workability, there was no need for the mechanical compaction of the layer to remove air pockets (Figure 7b). After the RPC hardened, its upper surface was moistened every few days for a month, and in the meantime, it was protected with PVC film. In the next stage, the testing of the beams was started, which is described in detail in the following chapter.

## 4. Description of Tests

### 4.1. Test Stand for the Loading of the Beams. Methods of Measuring the Beams’ Deflection, Strains, and Curvature

**Figure 8:**
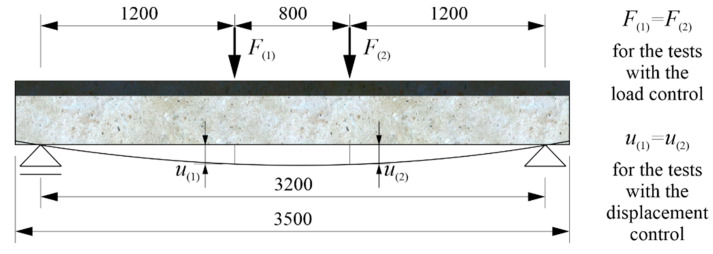
Static scheme of the beams’ loading ($F_{\left(1\right)}$, $F_{\left(2\right)}$ —loads from the actuators; $u_{\left(1\right)}$, $u_{\left(2\right)}$ —deflection under the actuators; dimensions in mm).

The same four-point bending scheme was adopted for each of 3 beams, as illustrated in Figure 8 for the strengthened beam.

**Figure 9:**
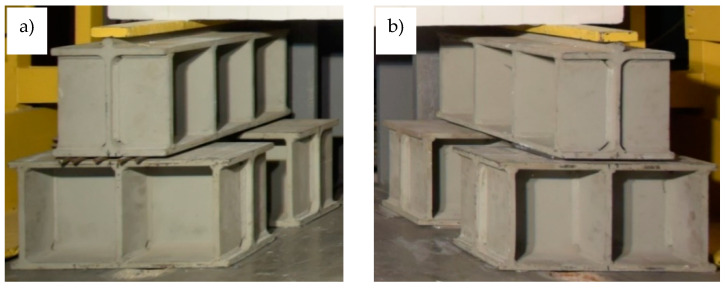
Beam supports: (**a**) roller support, (**b**) pinned support.

The supports’ axes were adopted at a distance of 15 cm from the ends of the beams; hence, the span was 320 cm. The loads from the actuators were applied symmetrically at a distance of 120 cm from the supports. During the loading cycles that did not lead to the reaching of the load-bearing capacity of the beams, the load values were controlled so that $F_{\left(1\right)}=F_{\left(2\right)}$. In the loading cycles leading up to reaching of the beams’ bending resistance, deflections were controlled so that $u_{\left(1\right)}=u_{\left(2\right)}$. According to the adopted static scheme, the free rotation of the beam at the axes of both supports was made possible by placing the beam using a flat bar on round bars welded to the bases (Figure 9a,b). In turn, to allow the left support to move freely, it was placed on the rollers (Figure 9a).

**Figure 10:**
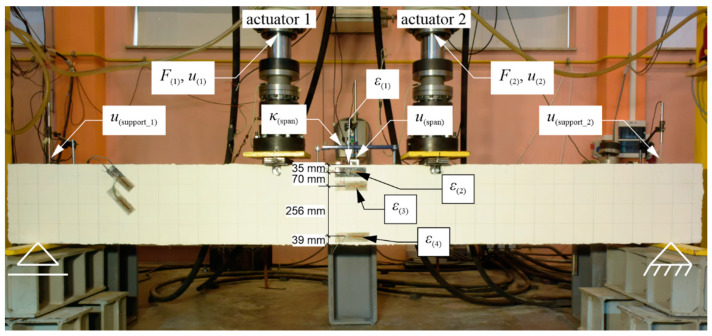
View of beam #1 on the test stand showing the places where the measurements were made (description of the symbols in the text).

The measurements of the selected quantities were carried out in the same way on each of the beams. For example, Figure 10 shows beam #1 after the installation of the measurement devices. The following were measured: the loads from the actuators ($F_{\left(1\right)}$ and $F_{\left(2\right)}$); the deflections at the point of load application ($u_{\left(1\right)}$ and $u_{\left(2\right)}$), the deflections at the middle of the span ($u_{\left(\mathrm{span}\right)}$) and the axis of the supports ($u_{\left(\mathrm{support}\_1\right)}$ and $u_{\left(\mathrm{support}\_2\right)}$); the normal strains along the beam axis at the middle of span on the upper surface of the beam ($\varepsilon_{\left(1\right)}$), at the level of the longitudinal upper rebars ($\varepsilon_{\left(2\right)}$), 10.5 cm from the upper surface ($\varepsilon_{\left(3\right)}$), and at the level of the longitudinal lower rebars ($\varepsilon_{\left(4\right)}$); and the curvature of the upper beam surface at the middle of the span ($\kappa_{\left(\mathrm{span}\right)}$). The symbols of these quantities are given in the parentheses. These symbols are also shown illustratively in Figure 10 at the relevant devices and sensors that measured them.

**Figure 11:**
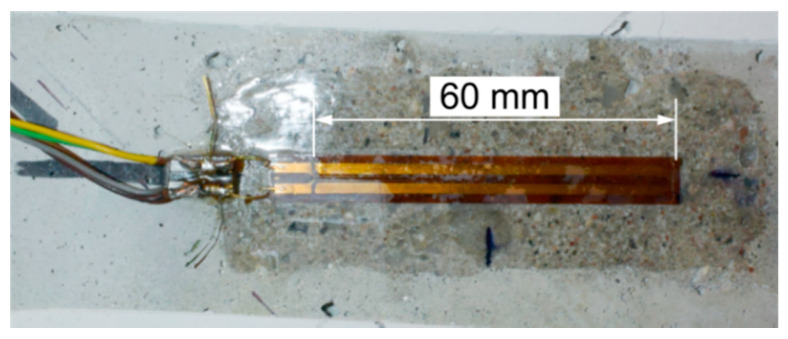
View of the glued strain gauge.

To measure the normal strains $\varepsilon_{\left(2\right)}$, $\varepsilon_{\left(3\right)}$, and $\varepsilon_{\left(4\right)}$, two strain gauges were used, which were attached symmetrically on vertical surfaces on both sides of the beam. All of the strain gauges had a resistance of 120 Ω and an active length of 60 mm (Tenmex TFs-60/120). They were glued with cyanoacrylate adhesive to previously-sanded and epoxy-impregnated surfaces of the concrete (Figure 11). Figure 10 also presents unmarked and diagonally glued strain gauges on the left side of the element, which were not used in further studies.

**Figure 12:**
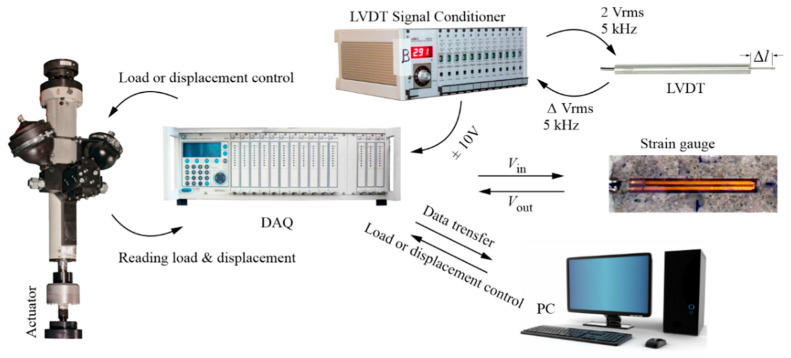
Configuration of the measurement chains for deflection, strains, and curvature.

All of the displacement measurements (except for the travel positions of the actuators, which were measured using sensors built in by their manufacturer) were performed using linear variable differential transformers (LVDTs; Pelltron PT×30) connected to the Pelltron MPL512 conditioner, which allowed us to generate an alternating voltage (in the range ±10 V) of a frequency of 5 kHz, and to read out the voltage differences as a result of the change in the position of the transducer core relative to the coils. The curvature was indirectly determined by means of the LVDT placed in a specially prepared device measuring the difference in the deflection of the upper beam surface along the section between the device supports (placed at a distance of 400 mm from each other) and the center of its span [54]. Then, using the equation of the circle inscribed in the line of the locally measured deflection along the section, the curvature of the beam’s axis at the middle of its span was approximated [54]. Before starting the measurements, each of the LVDTs’ measurement chains was calibrated separately, with an accuracy of 0.001 mm in the measured displacements. All of the sensor data listed above was recorded with frequency 20 Hz using the HBM MGCplus data acquisition system, a computer, and the Catman software. The configuration of the measurement chains is illustrated in Figure 12.

### 4.2. Method of Loading the Beams

**Figure 13:**
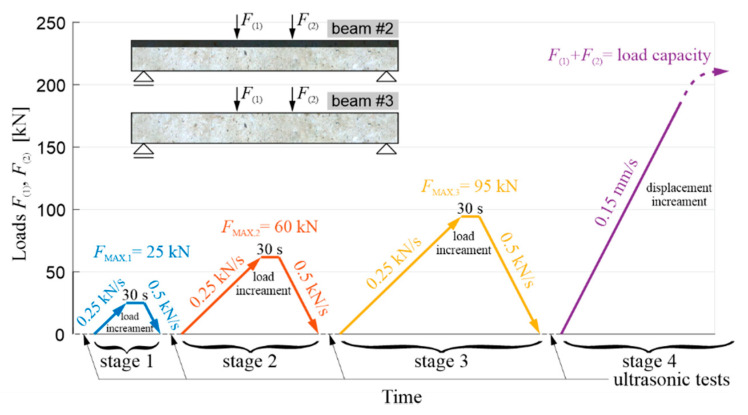
Procedure for the cyclic loading of beams #2 and #3, and ultrasonic testing.

First of all, the load-bearing capacity of the beam with the RPC strengthening was determined experimentally, verifying the correctness of the initial assumptions made. Beam #1 was loaded in one stage. The load was applied by controlling the displacement of the actuators’ pistons, which was increased at a constant speed of 0.15 mm/s. It was determined that the beginning of the yielding of the lower main reinforcement was achieved with a bending moment of about 236 kNm (i.e., with an average load of about 197 kN per actuator). Thus, the load-bearing capacity was about 10% higher than expected. Based on Equation (6), it can be concluded that this is primarily the effect of the increased actual yield point of the reinforcing steel in relation to the value minimally declared by the manufacturer. Based on this result, a cyclic load course was assumed in the comparative tests of beam #2, with the RPC layer, and reference beam #3, where the maximum load in a given cycle was increased from stage to stage. Three stages were adopted, with a maximum load of $F_{\mathrm{MAX},i}=$ 25 kN, 60 kN, 95 kN per the actuator in the sequence and in the fourth last stage until the beam was destroyed (or the maximum safe travel range of the actuators’ pistons was reached). The first three load levels thus corresponded to approximately 12.5%, 30%, and 50% of the load-bearing capacity of the RPC-strengthened beam, respectively. The load application course is shown in Figure 13. In stages 1–3, the load was applied by controlling the force (increase at 0.25 kN/s and decrease at 0.5 kN/s), with the maximum load in a given cycle being maintained for 30 s, and decrease F continued until the load was fully released. In the last stage, the load was applied by controlling the displacement of the actuators’ pistons, which was increased, as in the case of beam #1, at a constant speed of 0.15 mm/s. As mentioned in Section 1, this procedure was implemented in order to technically enable the ultrasonic testing between the different load stages (Figure 13).

### 4.3. Ultrasonic Testing

**Figure 14:**
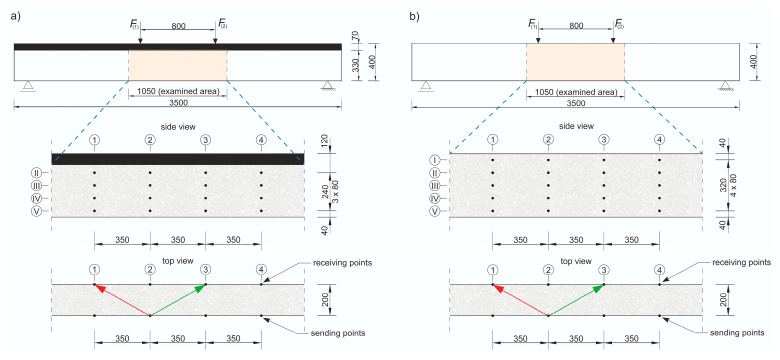
The areas of the beams for which the ultrasonic tests were carried out, with the scheme of the distribution of the sending and receiving points (Roman numerals indicate the rows of the points in horizontal planes, and Arabic numerals indicate the numbers of the successive points in given rows; dimensions are given in mm): (**a**) beam #2, (**b**) beam #3.

Ultrasonic testing is one of the most popular methods of the non-invasive diagnosis of materials and construction elements, including concrete, where information about their technical condition results from the speed analysis (e.g., [42,43,55,56]) and phase or amplitude changes (e.g., [57,58]) of the registered ultrasonic waves. Within the framework of the presented studies, it was decided to use the measurements of longitudinal wave velocity as a relatively easily-measurable and interpretable value in the case of a simplified adoption of the isotropic damage evolution model, according to the damage mechanics concept (e.g., [59]). Then, between the longitudinal wave velocity and the modulus of the elasticity of the material undergoing elastic degradation, there is the relationship (e.g., [43]):

(8) $$\frac{E_{D}}{E_{0}}={\left(\frac{c_{\mathrm{LD}}}{c_{L0}}\right)}^{2},$$

where $E_{0}$ and $E_{D}$ are the dynamic modulus of elasticity [Pa] of the material in an undamaged and damaged state, respectively; and $c_{L0}$ and $c_{\mathrm{LD}}$ are the longitudinal wave velocities [m/s] in the undamaged and damaged material, respectively. To apply Equation (8) directly, $c_{\mathrm{LD}}$ must be measured in the element damaged at the same level throughout its volume, e.g., under pure tension. In an RC beam under pure bending conditions, with the progress of the elastic degradation of the concrete, $E_{D}$ changes along the height, so Equation (8) can be used with the following simplifications: it is assumed that the state of deformation in the beam can be described by means of the hypothesis of the maintenance of the planarity of its cross-sections (as in the Bernoulli–Euler beam theory); the wave velocities are measured in the horizontal planes of the beam and, in their case, the relative change of Young’s modulus is estimated; and the influence of uneven distribution $E_{D}$ along the cross-section height on the curvature of the fastest propagation paths is negligible. Furthermore, the presence of the reinforcement causes the determined value of the $E_{D}/E_{0}$ relationship to be disturbed for this reason [56], which dictates the necessity to carry out the measurements, as far as possible, not along the longitudinal rebars or stirrup legs. In the case of bending, this makes the results obtained from Equation (8) approximate but useful in estimating the progress of the elastic concrete degradation. In order to be able to use Equation (8) to estimate the development of brittle beam damage, measurements were carried out in the middle section of the beam with a constant bending moment, and a system of sending and receiving points was used as in Figure 14.

The tests were carried out four times on beam #2 and #3 before each stage of beam loading (Figure 13). The time of the longitudinal wave propagation was measured using the ultrasonic pulse velocity instrument Punditlab between pairs of the sending and receiving points situated in horizontal planes, with an offset of one point diagonally to another (Figure 14). The 54 kHz transducers were used, which were selected to meet the basic requirements of ASTM [60] concerning the possibility of longitudinal wave generation, i.e., that five times the dominant wavelength is not greater than the transverse dimension of the element, and that the wavelength is not less than three times the average diameter of the aggregate particles. The measured average velocities for all of the stages were in the range 2804–3447 m/s (see Section 5.2 for details), which allowed us to meet these requirements. Five rows (No. I-V) with five sending and receiving points were used for beam #3, and four horizontal rows (No. II-V) with the same number of sending and receiving points per row were used for beam #2 with RPC strengthening (Figure 14). In beam #2, the use of the upper raw (No. I) in the RPC layer was abandoned due to the fact that the generation of the longitudinal waves in the 7 cm layer required the use of much higher frequency heads, but it was impossible to receive them effectively because of the increased damping caused by the steel fibres.

### 4.4. Photogrammetric Analysis

**Figure 15:**
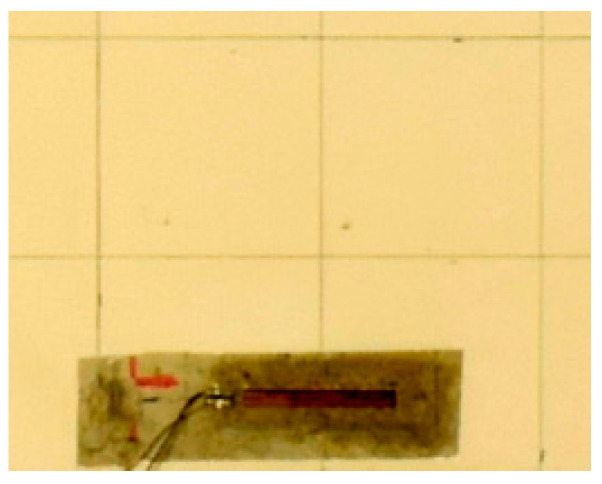
Example of a surface finishing detail on beam #1.

During the process of the loading of the beams, photographs of the beams were taken with a NIKON 1 v3 digital camera (resolution: 18 megapixels) at regular intervals (every 2 s) to archive the development of cracks. The obtained photos were subjected to photogrammetric analysis using an original computer program in C++, where algorithms were proposed in order to minimise user participation and automate the detection of cracks in the photos. The basis for the photogrammetric measurements proposed in the paper was the assumption of a significant difference in brightness recorded in the photo between the cracks on the surface of the beam and the surface of the beam itself. Before starting the loading, in order to enhance this difference and to equalize the brightness of the side surfaces of the beams, they were covered with a thin layer of white gypsum plaster (except for the strain gauges), which was then polished, and the beam was illuminated with a spot light source. Additionally, a positioning square grid with a 10 cm step (Figure 10 and Figure 15) was applied to facilitate the tracking of the brittle beam damage during the loading.

The camera was placed in front of the examined beam, at the minimum distance for which the whole side surface was visible in the photo, and, at the same time, so that the central part of the beam was in the central part of the photo. The position of the camera was constant during the whole process of loading the beam. The obtained series of photographs were analysed using an original algorithm implemented in a computer program. The principle of the algorithm’s operation is presented below.

**Figure 16:**
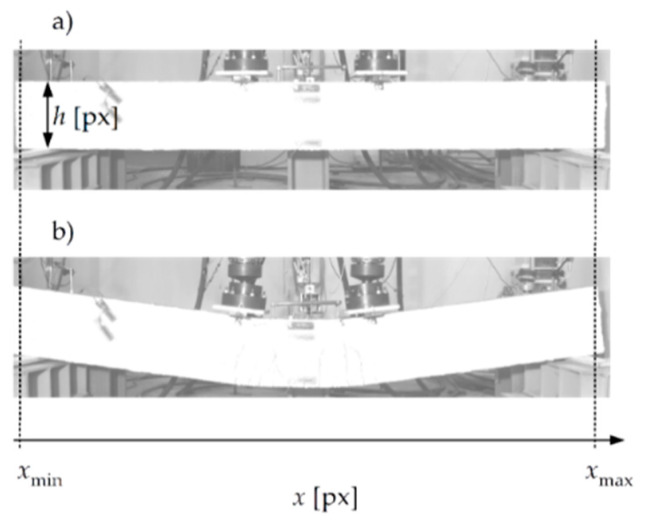
Reading  h, $x_{\min}$, and $x_{\max}$ from a series of beam testing photos: (**a**) the first photo of the unloaded beam, (**b**) the photo at the maximum beam’s deflection.

In the first stage, the area containing the side surface of the beam had to be determined automatically in the photos. At the beginning, the proposed algorithm requires the user to enter only three numbers: h, $x_{\min}$, and $x_{\max}$. These numbers are obtained by analysing two photos from the registered series: the first photo, with the beam’s zero deflection (Figure 16a), and the last photo, with its maximum deflection (Figure 16b). The height of the beam, h, is indicated in pixels in the first photo, whereas $x_{\min}$ and $x_{\max}$ are determined from the simultaneous analysis of both photos, also in pixels (Figure 16). They express, respectively, the minimum and maximum horizontal coordinates x of the vertical line, which simultaneously passes through the upper and lower edge of beam in both images.

**Figure 17:**
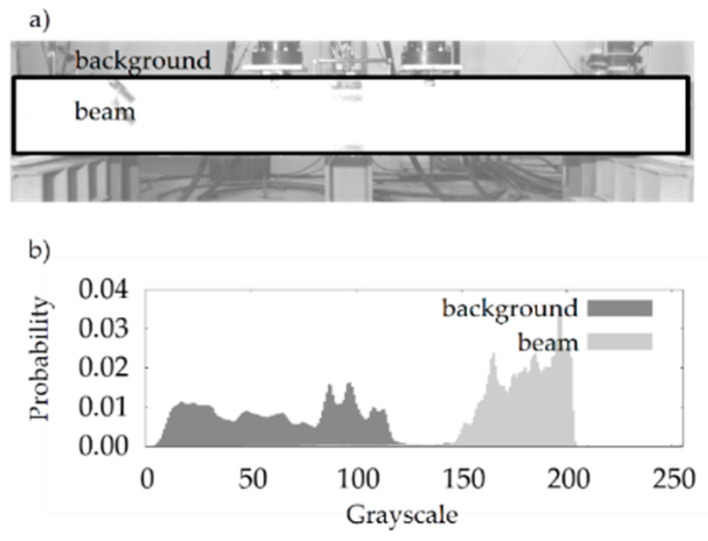
Divergent colour spectrum of the background and side surface of the beam in a grey scale: (**a**) sample photo of beam #1, (**b**) colour spectrum of the photo, with a division into background and beam surface.

Thanks to the applied finishing and lighting, the algorithm for the determination of the surface area of beam is based on the assumption that it is statistically the brightest element of each photo after conversion to black and white (Figure 17).

**Figure 18:**
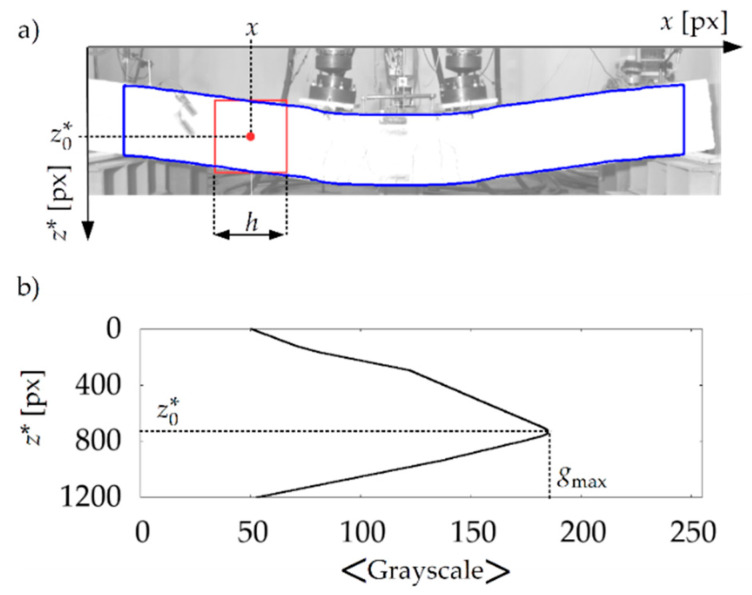
The method of detecting the side area of beam in the photo: (**a**) an example of the photo of beam #1, where the blue line marks its side area, as found by the algorithm; (**b**) the change of the average brightness of the squares with a side of h in a vertical direction for the x marked in the photo. The brightest point $z_{0}^{*}\left(x\right)$, where $\mathrm{Grayscle}=g_{\max}$, is approximately a point at the center of the beam’s height, and points in the range $(z_{0}^{*}-h/2,z_{0}^{*}+h/2$ ) are assigned to the side of beam.

On this basis, the pixels lying at the middle of the beams’ height along their length were found in the photographs. For this purpose, for each x of the range $\left(x_{\min}+h/2,x_{\max}-h/2\right)$, a pixel was found with the vertical coordinate $z_{0}^{*}\left(x\right)$, for which the mean value of brightness calculated in the square with center $\left(x,z_{0}^{*}\right)$ and side h was the highest. Therefore, for a given x, all of the points in the range $(z_{0}^{*}-h/2,z_{0}^{*}+h/2$) were considered to be the surface of the beam. The way in which this part of the algorithm works is illustrated in Figure 18.

**Figure 19:**
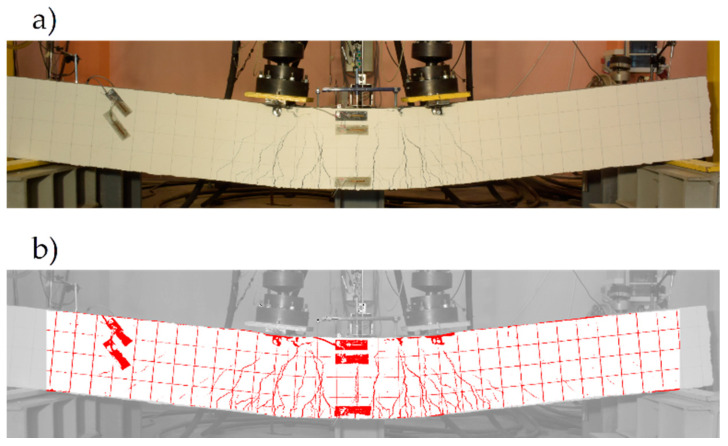
Illustration of the results of the algorithm used in the photogrammetric analysis: (**a**) a photo of cracked surface of beam #1; (**b**) an image of the beam after processing the photo with a visible map of the cracks. The areas representing the undamaged surface of the beam were marked in white, and the areas statistically deviating from the undamaged surface were marked in red.

In the second stage, the algorithm searches the pixels in which cracks are formed in the area assigned to beam. The observation that the cracks on the surface of beam have a significantly lower brightness than the undamaged surface of the beam was used. However, testing the brightness statistics for the entire beam’s surface and determining the border between the brightness of the undamaged surface and the brightness of the cracks on this basis gave unsatisfactory results. The main factor distorting the result was the brightness gradient on the beam’s surface (the areas further away from the light source had a lower brightness compared to areas closer to the light source). In order to eliminate this factor, it was proposed that the determination of beam’s surface brightness statistics should be based on the data from its square fragments. The parameters of these fragments were defined once, based on the analysis of the first photo from the series. In this photo, the position of the rectangle *R* was determined, which coincided with the visible side of the not-deflected beam, by stating in pixels its upper left-hand corner $\left(x_{c},z_{u}^{*}\right)$, width Δx, and height $\Delta z^{*}$. The algorithm then goes on to analyse the pixel brightness of the squares with a side of k, starting from k=10 pixels to k=h in pixels, which were generated in the *R* rectangle. The result was a value of $k=k_{0}$, for which the smallest variance was obtained from the width of the histograms describing the statistics of pixel brightness in all of the squares of *k* width within the R rectangle. The average width of the histograms $w_{0}$ was determined for $k_{0}$. The detection of the cracks in the subsequent photos was carried out as follows: (i) each pixel with $\left(x,z^{*}\right)$ coordinates belonging to the beam area is surrounded by a square with a side of $k_{0}$ and its centre at point $\left(x,z^{*}\right)$; (ii) for pixels of the square lying simultaneously in the beam area, a brightness histogram is generated, and the average brightness g is calculated from it; (iii) if the brightness at point $\left(x,z^{*}\right)$ is less than $g-3w_{0}$, this point is marked in red (as belonging to a crack), and if not, it is marked in white. Due to the darker colours of the positioning grid and the measuring instrumentation, the algorithm also assigned red to the pixels in this case. For example, the result of the algorithm for beam #1 is shown in Figure 19.

However, knowing the location of these elements, it is possible to easily distinguish them from the cracks and mark them using a different colour, which was performed when presenting some of the results in the further part of article. The advantage of the presented algorithm is the possibility of the ‘automatic’ analysis of a large number of photos, which allows us to analyse the evolution of the cracks over time.

## 5. Research Results and Discussion

### 5.1. Beam #1

**Figure 20:**
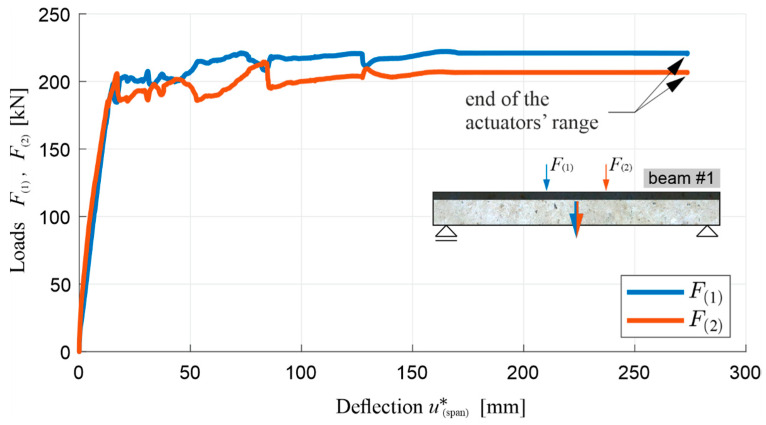
Load diagram in the function of the deflection at the middle of the span of beam #1, with the equal displacement of the actuators being forced.

Figure 20 shows, for beam #1, a diagram of the loads $F_{\left(1\right)}$ and $F_{\left(2\right)}$ (respectively from actuators 1 and 2, visible in Figure 10) as a function of $u_{\left(\mathrm{span}\right)}^{*}$, i.e., the corrected deflection at the middle of the span determined in relation to the actual support position:

(9) $$u_{\left(\mathrm{span}\right)}^{*}=u_{\left(\mathrm{span}\right)}-\frac{u_{\left(\mathrm{supprot}\_1\right)}+u_{\left(\mathrm{support}\_2\right)}}{2}.$$

It can be seen in the graph that the control of displacements $u_{\left(1\right)}=u_{\left(2\right)}$ imposed in this case means that the loads $F_{\left(1\right)}$ and $F_{\left(2\right)}$ are not equal, due to the impossibility of making a perfectly symmetrical system in reality. However, $F_{\left(1\right)}$ and $F_{\left(2\right)}$ deviate so slightly that, in the middle section of the beam, the bending moment is roughly a constant function. Nevertheless, the load values presented in the further part of the article—on diagrams or in numbers—are presented as totals, i.e., $F_{\left(1\right)}+F_{\left(2\right)}$ (including beams #2 and #3, the research on which is presented in Section 5.2). Beam #1 also demonstrated a very favourable behaviour in the post-critical state, and an increased energy absorption capacity. It is worth noting at this point that the use of a relatively thin layer of RPC enabled the full potential of the used reinforcing steel to be exploited, taking into account its increased ductility. For this reason, the test had to be stopped before the bending resistance of the beam was reached with deflection of 278.5 mm, i.e., when the maximum safe actuators’ travel range was reached. This fact is indicated in the diagrams below.

**Figure 21:**
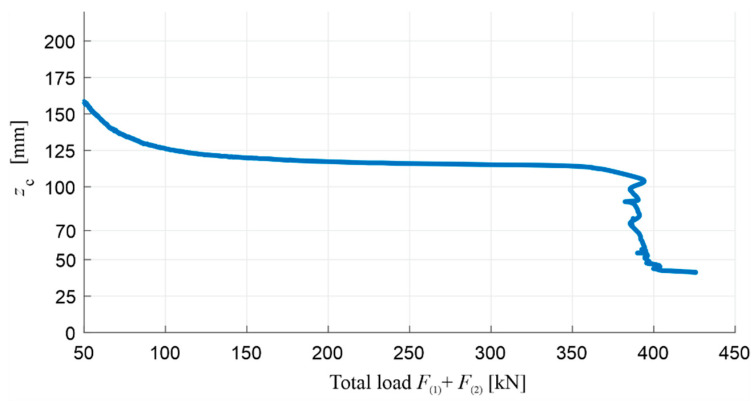
Diagram of the position of the neutral axis as its distance from the compression face of beam #1 at the middle of the span in the function of the total load.

Before interpreting the other results, the position of the neutral axis of normal stress at the middle of the beam span in the function of the load was first estimated as key information in order to evaluate the initial assumptions made in Section 3.1. The position of the neutral axis is shown in Figure 21 as $z_{c}$, i.e., as its distance from the compression face of the beam according to Figure 4a. Using the measurements of strains $\varepsilon_{\left(1\right)}$ and $\varepsilon_{\left(2\right)}$, i.e., respectively on the upper surface of the beam and at a distance of 35 mm from it (Figure 10), and additionally assuming that the state of the strains in the beam can be described according to the Bernoulli–Euler beam theory, the changes of $z_{c}$ were approximated from the relation:

(10) $$z_{c}=\frac{\varepsilon_{\left(1\right)}}{\varepsilon_{\left(1\right)}-\varepsilon_{\left(2\right)\mathrm{mean}}}\cdot 35\;\mathrm{mm},$$

where $\varepsilon_{\left(2\right)\mathrm{mean}}$ is the average strain of two strain gauges measuring $\varepsilon_{\left(2\right)}$. In the diagram, it can be seen that, after the initial cracking of the tension zone, the position of the neutral axis varies slightly, from approximately 125 mm to 110 mm from the top edge of the beam’s cross-section, and the compression zone covers both the OC and the RPC. In this respect, it can also be considered that the tension steel and compressed OC and RPC are in the linear range of the stress–strain relation. This stage is carried out in the load range from approximately 25% of the load-bearing capacity of the beam, until the lower rebars are yielded.

When the tensile stress in the lower rebars reaches the steel’s yield point, and the plastic flow process begins in them, the neutral axis starts to move to the top of the cross-section, and the load stops increasing significantly. It is worth noting that, by using RPC, the compression zone could only reach a height of about 40 mm while maintaining the stability of the structure of this material. In turn, when the neutral axis reaches the boundary between the OC and RPC layers ($z_{c}=70$ mm), $\varepsilon_{\left(1\right)}\approx -0.0023$ was measured, which allows us to estimate, with Equation (4), the normal strain in the lower reinforcing steel as $\varepsilon_{s\;\mathrm{LS}}\approx 0.0096$. On the other hand, the bending moment at the middle of span at this moment was $M_{\left(\mathrm{span}\right)}\approx 233.5$ kNm, which, assuming a stress distribution similar to that in Figure 4a, allows us to estimate the value of maximum stress in the RPC layer as:

(11) $$\left|\sigma_{c\;\mathrm{LS}}\right|=\frac{2}{bh_{\mathrm{RPC}}}\;\frac{M_{\left(\mathrm{span}\right)}}{d-\frac{h_{\mathrm{RPC}}}{3}}\approx 99\;\mathrm{MPa},$$

The value of $\varepsilon_{s\;\mathrm{LS}}$ is in the range $\left(f_{y}/E_{s},\varepsilon_{u}\right)=\left(0.0025,0.08\right)$ and $\left|\sigma_{c\;\mathrm{LS}}\right|\approx 0.5\cdot f_{c\;\mathrm{RPC}}$, where $f_{c\;\mathrm{RPC}}$ is taken as 197.9 MPa, based on Table 3. Therefore, the basic assumptions adopted in Section 3.1 concerning the beam’s ultimate bending limit state, and the calculations of its optimum reinforcement ratio based on it, were fulfilled. The moment when the neutral axis reaches the layer boundary is also illustratively marked on the subsequent figures, which show the deflection, curvature, and strains of beam #1.

**Figure 22:**
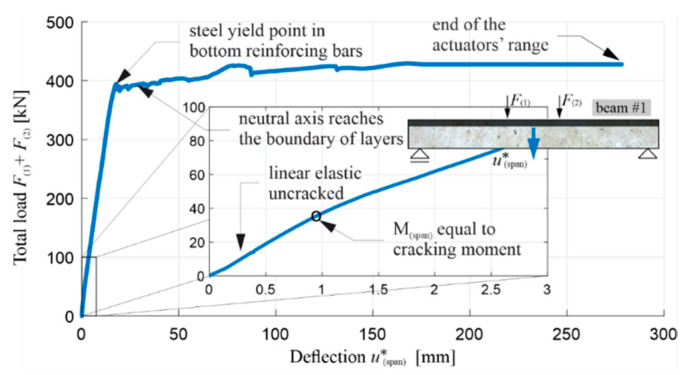
Diagram of the total load in the function of the deflection at the middle of the span for beam #1.

**Figure 23:**
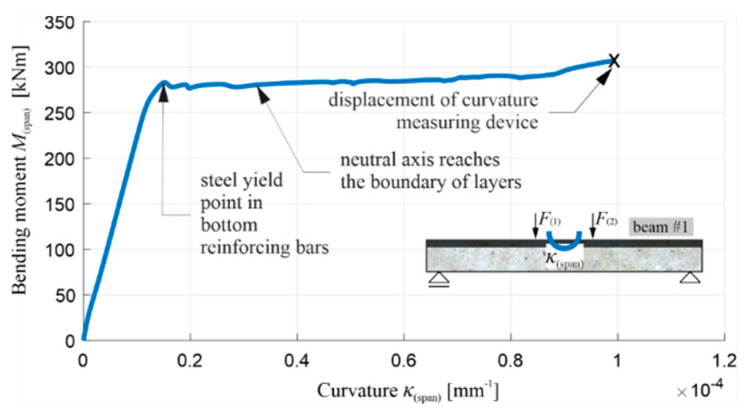
Diagram of the bending moment in the function of the curvature at the middle of the span for beam #1.

Figure 22 shows a diagram of the total load $F_{\left(1\right)}+F_{\left(2\right)}$ in function of deflection $u_{\left(\mathrm{span}\right)}^{*}$, with the initial part of the diagram enlarged, and Figure 23 shows a bending moment diagram at the middle of span $M_{\left(\mathrm{span}\right)}$ in the function of curvature $\kappa_{\left(\mathrm{span}\right)}$ at the middle of the span. The diagram $M_{\left(\mathrm{span}\right)}$ vs $\kappa_{\left(\mathrm{span}\right)}$ was finished earlier than the diagram $F_{\left(1\right)}+F_{\left(2\right)}$ vs $u_{\left(\mathrm{span}\right)}^{*},$ due to the fact that the curvature measuring device was moved uncontrolled during the study, which was marked with an x. From Figure 22 and Figure 23, it is possible to determine the moments of the initiation of the cracks in the OC (when the bending moment in the middle of the span equals the cracking moment) and the beginning of the yielding of the lower reinforcing steel, and to confirm the stable behaviour of the beam in the phase after the yielding of the tension steel reinforcing bars.

It is possible to read from the diagrams, the values of the total load, the bending moment, the deflection, and the curvature of beam’s axis, at the moments of the first cracks appearing, the beginning of the yielding of the reinforcing steel, the reaching of the boundary of beam’s layers by the neutral axis, and at the end of test, as summarized in Table 5. Table 5 does not indicate the curvature of the beam at the end of test, and instead indicates this by the symbol ND (not determined) for the reason already mentioned.

**Figure 24:**
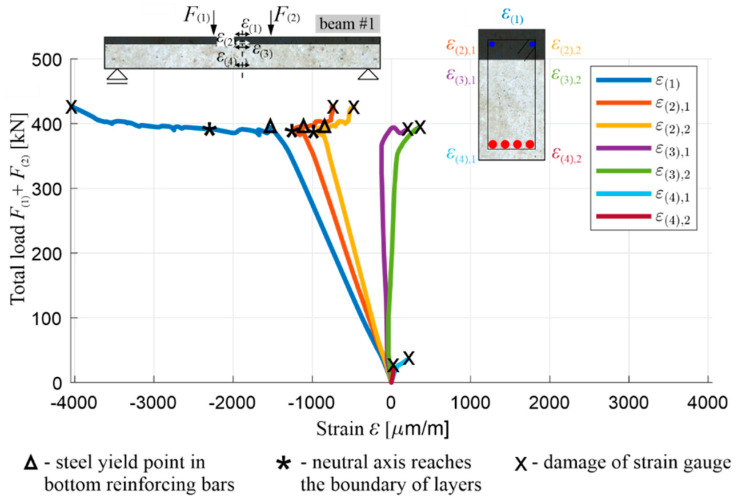
Diagram of the total load in the function of the measured strains at the middle of the span for beam #1.

Figure 24 shows the course of the total load $F_{\left(1\right)}+F_{\left(2\right)}$ in the function of the measured normal strain $\varepsilon_{\left(1\right)}$-$\varepsilon_{\left(4\right)}$ along the axis of the beam, with two separate diagrams for $\varepsilon_{\left(2\right)}$-$\varepsilon_{\left(4\right)},$ due to the fact that the strain gauges were applied in this case to both sides of the beam’s cross-section. The moments when the strain gauge or the concrete cover (to which it was glued) were damaged are marked with an x. These diagrams confirm the conclusions of the diagrams in Figure 21, Figure 22 and Figure 23. The measurement of $\varepsilon_{\left(4\right)}$ at the level of the lower reinforcement shows the moment when the first cracks appear in the tension OC at the same load level, as shown in Figure 22. The strain gauges measuring $\varepsilon_{\left(4\right)}$ were damaged at the value of $M_{\left(\mathrm{span}\right)}$ of the order of cracking moment. In turn, the measurement of $\varepsilon_{\left(3\right)},$ just below the layers’ boundary, shows the moment of the reaching of the normal stress neutral axis the position of these strain gauges, just before the lower reinforcing steel is yielded. The courses of $\varepsilon_{\left(1\right)}$ (on top of the beam) and $\varepsilon_{\left(2\right)}$ (at the middle of the height of upper layer) also show that the RPC in the vicinity of these points remained in compression throughout the test. The flat part of the diagrams of $\varepsilon_{\left(1\right)}$ and $\varepsilon_{\left(2\right)}$, at the top of the diagrams, shows that, after the lower reinforcing steel has been yielded, and the load and bending moment levels have been stabilized, the strains continue to increase as a result of the increasing curvature (Figure 23). With the simultaneous reduction of concrete compression zone (Figure 21), this indicates an increase in the stress in the RPC, such that the bending moment remains approximately constant. This increase is stable as long as the stresses in the RPC do not approach the compressive strength, causing it, at this moment, to be partially crushed and loosened in the near-surface part not encircled by the stirrups.

**Figure 25:**
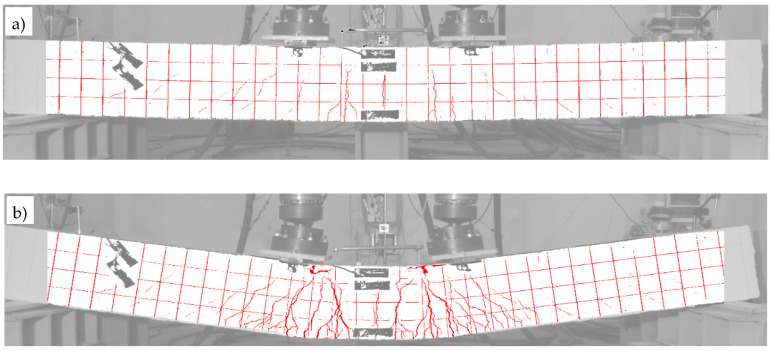
Views of the cracking of beam #1: (**a**) the neutral axis reaches the layer boundary according to Equation (10) ($z_{c}=$ 7 cm); (**b**) the actuators reach the maximum travel range. In order to increase the clarity of the figure, the instrumentation and the places where the strain gauges were glued are painted in grey, and the positioning grid (10 cm mesh) and the cracks are marked in red.

The fact of the partial spalling of the RPC and the crushing of this layer already in the final phase of the test was recorded by means of photogrammetric tests carried out according to the procedure described in Section 4.4. The obtained results are presented in Figure 25, where two characteristic moments of the test are illustrated, i.e., when the neutral axis of the normal stresses reached the boundary of the OC and RPC layers (Figure 25a), and at the end of the test, when the actuators’ pistons reached the maximum safe range of their travel (Figure 25b). The first picture was chosen for a moment that corresponds to $z_{c}=h_{\mathrm{RPC}}=70$ mm, according to Figure 21, which indirectly confirms the correctness of the use of Equation (10) in the presented deliberations, and the correctness of the strain measurements. At this moment, the top ends of the cracks reach furthest just before the border of the OC and RPC layers, and the RPC layer behaves in a stable fashion. The cracks have a maximum width of 2.1 mm at this moment. In turn, in Figure 25b, at the end of the test, two plastic hinges underneath the actuators and the first damage in the RPC layer can be clearly seen. The cracks already had a maximum width of 6 mm.

### 5.2. Beams #2 and #3

Beam #2 (with the RPC layer), similarly like beam #1, also displayed a very beneficial behaviour in the post-critical state, and an increased ability to absorb energy. For this reason, the tests for beam #2 had to be interrupted with a deflection of 178.5 mm when the maximum safe travel range of the actuators’ pistons was reached. In turn, in the case of beam #3 (made entirely of the OC), it was possible to exhaust its bending resistance at a deflection equal to 71.5 mm, where the direct cause of its destruction was the crushing of the compression zone and the extensive delamination of the top concrete cover. This information is also described in the diagrams and tables below.

**Figure 26:**
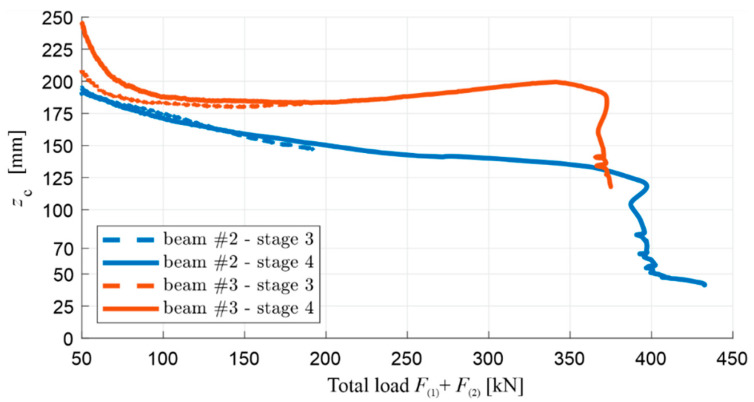
Diagram of the position of the neutral axis as its distance from the compression face of beams #2 and #3 at the middle of the span in the function of the total load in stages 3 and 4 during the increasing of the load.

The position of the normal stress neutral axis in the function of its distance $z_{c}$ from the compression face of both beams is shown comparatively in Figure 26. $z_{c}$ was calculated from Equation (10) for the last two loading cycles (stage 3 and stage 4) using the fact that the localization of strain gauges was identical to that of beam #1. In the diagram, it can be seen that the position of the neutral axis stabilises after the cracking of the beams’ lower tension zone, which happened similarly for beam #1. It is in the load range from about 25% of the load-bearing capacity until the yielding process of the lower reinforcement steel begins. In this stage for beam #2, $z_{c}$ changes from approximately 175 mm to 125 mm, and $z_{c}$ varies between approximately 185 mm and 200 mm for beam #3. Thus, the height of the compression zone in beam #3 is greater, and, just before the load-bearing capacity is exhausted, it is about 60% greater than the height of the compression zone in beam #2. This difference is clearly due to the higher stiffness under compression of the RPC layer in beam #2, compared to the OC creating the entire compression zone in beam #3. After the lower rebars are yielded, the load ceases to increase significantly, and the neutral axis begins to approach the top edge of the cross-section quickly. The compression zone was crushed at $z_{c}\approx 120$ mm in beam #3, while the height of the compression zone in beam #2, similarly to beam #1, was reduced while maintaining the stability of the structure of this material up to about 40 mm.

In the case of beam #2, when the neutral axis reaches the border between the OC and RPC layers ($z_{c}=70\;$ mm), $\varepsilon_{\left(1\right)}\approx -0.0025$ was measured, which corresponds to the normal strain in the lower rebars $\varepsilon_{s\;\mathrm{LS}}\approx 0.0104,$ according to Equation (4). At the same time, the maximum stress in the RPC layer, as estimated on the basis of Equation (11), is $\left|\sigma_{c\;\mathrm{LS}}\right|\approx 101$ MPa, with a span bending moment of $M_{\left(\mathrm{span}\right)}\approx 238.4$ kNm. The results obtained, similarly to those for beam #1, meet the basic assumptions adopted in Section 3.1 concerning the ultimate limit of the bending state and the optimum reinforcement ratio determined on this basis. Namely, the value of $\varepsilon_{s\;\mathrm{LS}}$ is in the range $\left(f_{y}/E_{s},\varepsilon_{u}\right)=\left(0.0025,0.08\right)$, and $\left|\sigma_{c\;\mathrm{LS}}\right|\approx 0.51\cdot f_{c\;\mathrm{RPC}},$ where $f_{c\;\mathrm{RPC}}$ is taken as 197.9 MPa based on Table 3. It is worth noting, at this point, that the value of $z_{c}$ did not reach 70 mm during the reliable measurements of $\varepsilon_{\left(1\right)}$ and $\varepsilon_{\left(2\right)}$ in the case of beam #3, which were interrupted by the initiated concrete crushing process in the compression zone. The moment when the neutral axis reaches the layer boundary in beam #2 is also illustratively marked on the subsequent figures, which show the measured deflection, curvature, and strains.

**Figure 27:**
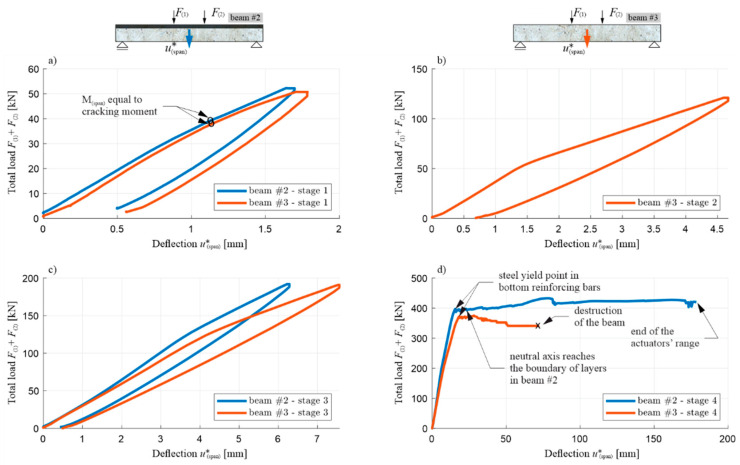
Diagram of the total load in the function of the deflection at the middle of the span for beams #2 (blue line) and #3 (red line): (**a**) stage 1, (**b**) stage 2, (**c**) stage 3, (**d**) stage 4.

**Figure 28:**
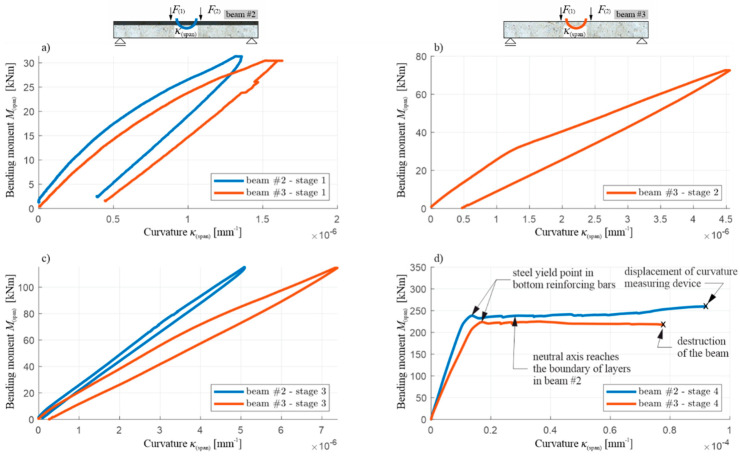
Diagram of the bending moment in the function of the curvature at the middle of the span for beams #2 (blue line) and #3 (red line): (**a**) stage 1, (**b**) stage 2, (**c**) stage 3, (**d**) stage 4.

Figure 27 and Figure 28 show—comparatively for beams #2 and #3 in individual loading cycles—a total load diagram $F_{\left(1\right)}+F_{\left(2\right)}$ in the function of the deflection $u_{\left(\mathrm{span}\right)}^{*}$ (Figure 27), and a bending moment diagram $M_{\left(\mathrm{span}\right)}$ in function of curvature $\kappa_{\left(\mathrm{span}\right)}$ (Figure 28) at the middle of span. In the case of beam #2, the diagram $M_{\left(\mathrm{span}\right)}$ vs $\kappa_{\left(\mathrm{span}\right)}$ was finished earlier than the diagram $F_{\left(1\right)}+F_{\left(2\right)}$ vs $u_{\left(\mathrm{span}\right)}^{*}$, due to the fact that the curvature measuring device was moved uncontrolled during the loading, which was marked with an x. The moment of the destruction of beam #3 is also marked with an x. The values shown for these quantities are to be treated as increments in each individual load stage, i.e., the sensor indications had been zeroed before the load application was started again. In addition for these diagrams, the displacement and curvature changes in stage 2 for beam #2 were not plotted out due to unforeseen disruption of the data transmission from the two LVDTs.

Similarly to the corresponding diagrams for beam #1, the moment of the initiation of the cracks in the tension zone of the OC can be determined for beams #2 and #3, based on Figure 27 and Figure 28 (when the bending moment at the middle of the span is equal to the cracking moment, i.e., with a total load of 38–39 kN in both beams) and the beginning of the yielding of the lower reinforcement steel (with a total load of 397.1 kN for beam #2 and 372.5 kN for beam #3). The use of a 7 cm thick RPC layer therefore increased the load, leading to the initiation of the yielding of the tension rebars by approximately 6%. Comparing the results obtained, it can be seen that, in each load stage, the recorded displacement and curvature are, for the same loads, higher in beam #3 compared to beam #2, which clearly shows the higher stiffness of the element with an RPC layer. For example, when the reinforcement steel starts to yield, the deflection of beam #2 is approximately 15% less than that of beam #3. It is also worth noting the convergence of the results for beam #1 and #2 with the RPC layers. Comparing Figure 22 with Figure 27d, and Figure 23 with Figure 28d, it can be seen that the yielding of the steel in the bottom reinforcing bars is achieved at $M_{\left(\mathrm{span}\right)}$ equal to 236.5 kNm and 238.3 kNm for beam #1 and #2, respectively. They are very similar despite the fact beam #1 was loaded in one stage and beam #2 in four cyclic stages. The low cyclic load applied to beam #2 did not, therefore, affect the increased development of the damage and the reduction of the load-bearing capacity in this case.

The total load–deflection (Figure 27) and moment–curvature (Figure 28) diagrams also show clearly, in loading stages 2–4, a sudden decrease in the slope of the curves when reaching the maximum load level from the previous stage, and a decrease in the slope of the sections during the unloading relative to the slope of the curve sections at the beginning of the load application in a given stage. This shows that the stiffness of the tested beams decreases as a result of the accumulation of brittle concrete damage, with the mechanisms of crack evolution being re-initiated only when the stress level—which was at its maximum in the preceding load stage—is reached. The curves of the load–deflection and moment–curvature relations do not form closed curves at full unloading, which also indicates the initiation of plastic deformations in the concrete, starting from stages 1–3, but they start growing in the subsequent loading stages, similarly to brittle damage, only after reaching the stress level which was the maximum in the previous stage.

The diagram in Figure 27d also shows significant differences in the behaviour of beams #2 and #3 in the post-critical state after yielding the bottom rebars. The difference in the maximum load achieved between beams #2 and #3 is about 12%, and it is greater for the beam with the RPC strengthening. The load increases slightly in beam #2 and decreases in beam #3 after the tension rebars have been yielded. Importantly, the same as in beam #1, the thin RPC layer caused the energy absorption capacity of beam #2 to be increased significantly, compared to the situation where only OC was used in the element. 

Table 6 gives a summary of the values (read from Figure 27 and Figure 28 for beams #2 and #3) of the total load, bending moment, deflection, and curvature of the beams’ axes at the middle of the span at the moments of the beginning of the concrete’s cracking in the tension zone, at the maximum loads in the subsequent load stages, and at the moment of the yielding of the lower rebars. Furthermore, the values of these quantities at the end of the last stage 4 are presented at the moment of the destruction of beam #3, and for beam #2 at the end of the travel range of the actuators’ pistons, for reasons mentioned in the introduction of this chapter. In addition, the values listed are shown for beam #2 when the neutral axis of the normal stress reaches the boundary between the RPC and OC layers. If some data could not be read, including because of a temporary sensor signal transmission interruption or a malfunction of the measuring device, Table 6 indicates this situation with the ND symbol.

**Figure 29:**
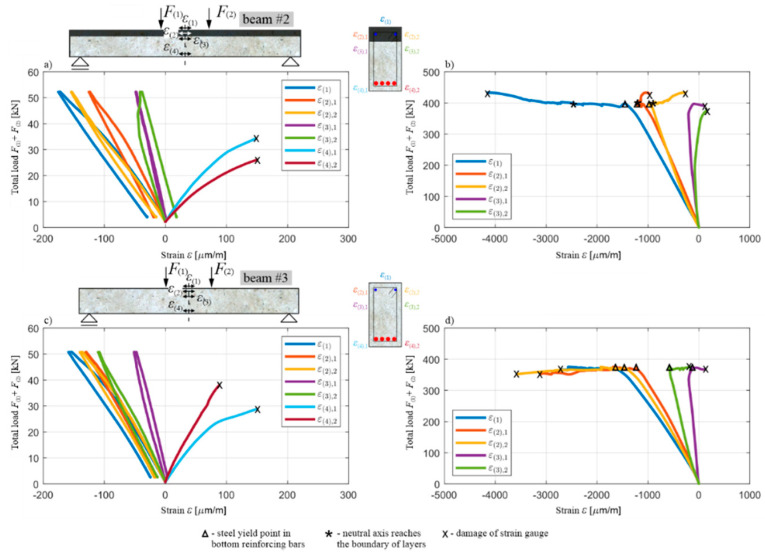
Diagram of the total load in the function of the measured strains at the middle of the span for beam #2 in stage 1 (**a**) and stage 4 (**b**), and beam #3 in stage 1 (**c**) and stage 4 (**d**).

Figure 29 shows the course of total load $F_{\left(1\right)}+F_{\left(2\right)}$ in the function of the measured normal strain $\varepsilon_{\left(1\right)}$-$\varepsilon_{\left(4\right)}$ along the axis of beams #2 and #3 in the first and fourth stage, with two diagrams separately for $\varepsilon_{\left(2\right)}$-$\varepsilon_{\left(4\right)}$ due to the fact that the strain gauges were applied in this case on both sides of the beams’ cross-section. The moments when the given strain gauge, or the concrete cover (to which it was glued) were damaged, are marked with x. In the case of strains $\varepsilon_{\left(4\right)}$, the strain gauges measuring them were damaged at $M_{\left(\mathrm{span}\right)}$ values of the cracking moment order (Figure 29a,c), as a result of the concrete cracking in their immediate vicinity. Comparing the strain values in stage 1, it can be seen that, already in this stage, the position of the neutral axis of normal stress differs in both beams, and it is placed higher in beam #2. This is evidenced by the higher values of compression concrete strain $\varepsilon_{\left(3\right)}$ in beam #3 measured at the same load compared to beam #2 (Figure 29a,c). In stage 4, the $\varepsilon_{\left(3\right)}$ measurement reveals in beam #2, similarly to beam #1, that the neutral axis level under loads leading to the yielding of the lower reinforcing steel crosses up the level of the strain gauges measuring the $\varepsilon_{\left(3\right)}$ strain (Figure 29b), which is not clearly observed in beam #3 (Figure 29d). During stage 4 (Figure 29b,d), it can also be seen, by comparing the changes of $\varepsilon_{\left(1\right)}$ (on the top of the beam) and $\varepsilon_{\left(2\right)}$ (at a distance of 3.5 cm from the top of the beam), that the steel yield point in the bottom reinforcing bars is achieved with lower compression concrete strain values for beam #2 than for beam #3, which again clearly confirms the higher stiffness of RPC compared to OC, and the resulting higher global bending stiffness for beam #2. Furthermore, it can be observed that the strain of $\varepsilon_{\left(2\right)}$ (Figure 29b,d) continues to increase in beam #3 after the yielding of the lower reinforcing steel, while it starts to decrease at some moment in beam #2 (similarly to beam #1—Figure 24), because of the possibility to reduce significantly the compression zone of the concrete in beam #2 by using RPC strengthening while simultaneously maintaining the stable structure of this material (compare with Figure 26).

**Figure 30:**
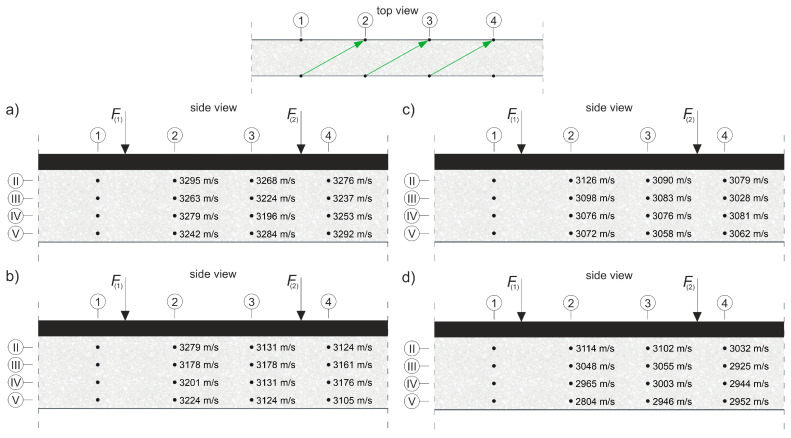
Velocities of the longitudinal waves in beam #2 propagating between the sending and receiving points situated diagonally with one point offset in the right direction, before: (**a**) stage 1, (**b**) stage 2, (**c**) stage 3, (**d**) stage 4.

**Figure 31:**
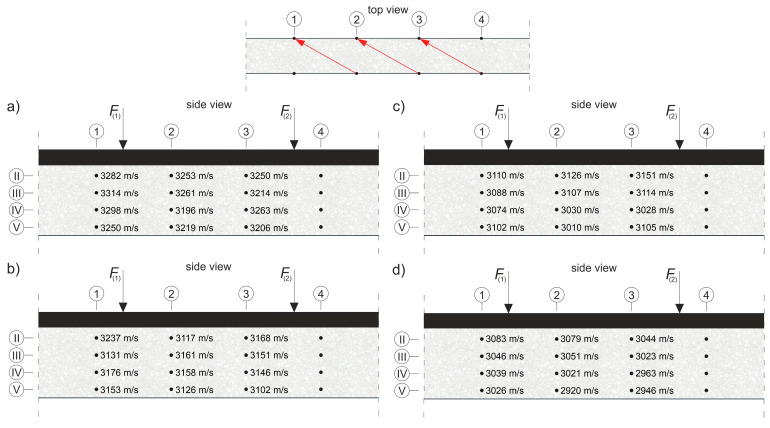
Velocities of the longitudinal waves in beam #2 propagating between the sending and receiving points situated diagonally with one point offset in the left direction, before: (**a**) stage 1, (**b**) stage 2, (**c**) stage 3, (**d**) stage 4.

**Figure 32:**
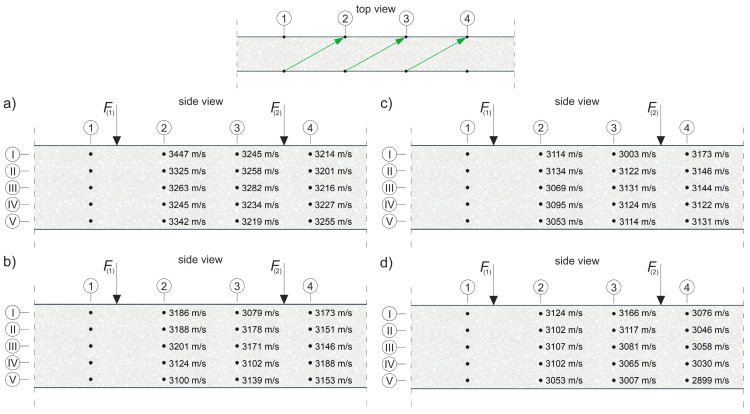
Velocities of the longitudinal waves in beam #3 propagating between the sending and receiving points situated diagonally with one point offset in the right direction, before: (**a**) stage 1, (**b**) stage 2, (**c**) stage 3, (**d**) stage 4.

**Figure 33:**
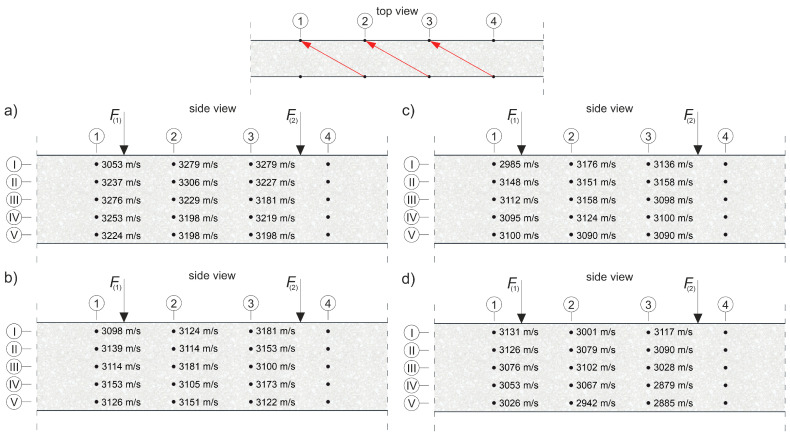
Velocities of the longitudinal waves in beam #3 propagating between the sending and receiving points situated diagonally with one point offset in the left direction, before: (**a**) stage 1, (**b**) stage 2, (**c**) stage 3, (**d**) stage 4.

The following Figure 30, Figure 31, Figure 32 and Figure 33 show the results of the ultrasonic tests carried out according to the procedure described in Section 4.3. The tests were carried out before each of the load stages, and their results are the velocity values of the longitudinal ultrasonic waves propagating between the sending and receiving points lying at an angle of 45° relative to each other at the same horizontal levels. The decreases in these velocities in the subsequent research stages indicate a brittle damage evolution in the concrete structure, contributing to the reduction of its stiffness. In the case of the testing of beam #2, it was not possible to effectively perform ultrasonic measurements for the points lying at the middle of RPC layer’s height, due to the dispersed steel fibre reinforcement used in its composition effectively damping the impulses.

**Figure 34:**
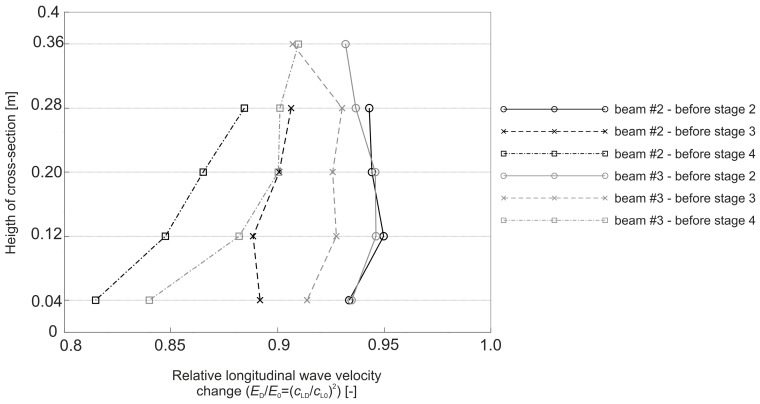
Summary diagram of the average changes of the velocity of the longitudinal waves along the height of the middle section of beams #2 and #3, calculated according to Equation (8).

Based on the measurements of the longitudinal wave velocities shown in Figure 30, Figure 31, Figure 32 and Figure 33, Figure 34 presents a diagram of the average dynamic changes of the Young’s moduli of the concrete along the height of the middle section of beams #2 and #3, estimated according to Equation (8). In the diagram shown in Figure 34, it can be seen that the relative stiffness decreases in the dynamic Young’s modulus relative to the virgin state are similar in both beam #2 and beam #3 between loading stages 1 and 2 (when the bending resistance of the beams was used in about 12.5%), and they are approximately 5–7%. In the case of the results obtained from the measurements between stages 2 and 3, and 3 and 4, there are differences in the stiffness change of beams #2 and #3. For example, the drops of the dynamic Young’s moduli are greater in beam #2 between stages 3 and 4 (when the load-bearing capacity of the beams was used in about 50%), and reach 12% and 19% at the top and bottom of the OC layer, respectively, and in beam #3 about 11% and 16% at the same points. After the subsequent loading stages, there is also an increasingly clear trend, resulting from the way in which the beams deformed under the bending and brittle properties of concrete, showing that the stiffness drops change along the height of the cross-section, increasing in the downward direction. Of course, this is the case here, where the resistance of the concrete compression zone in the top part of the beams’ cross-section has not yet been exhausted. The higher stiffness drops of the tension concrete in beam #2 are also confirmed by the fact—as previously found by the strain measurements—that the neutral axis of normal stress in beam #2 is situated higher than in beam #3, and that the lower reinforcing bars, when using the top layer of RPC, can be subject to higher elongations due to the significantly increased ability of RPC to transmit compressive stresses compared to OC.

The results presented in Figure 30, Figure 31, Figure 32, Figure 33 and Figure 34 also show the perspective possibilities of using relatively simple measurements of longitudinal wave velocity changes along a height of the beams in diagnostics of this type of structural elements, as well as the indirect evaluation of the degree of the utilisation of their bending resistance, and the tracking of the changes of the position of the neutral axis for normal stress.

**Figure 35:**
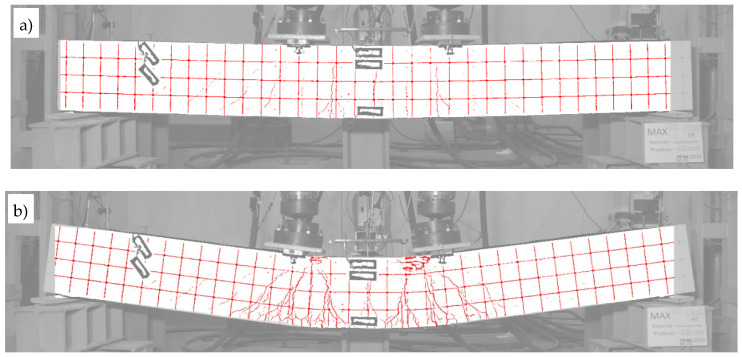
Views of the cracking of beam #2: (**a**) the neutral axis reaches the layer boundary according to Equation (10) ($z_{c}=$7 cm); (**b**) the actuators reach the maximum travel range. In order to increase the clarity of the figure, the instrumentation and the places where the strain gauges were glued are painted in grey, and the positioning grid (10 cm mesh) and cracks are marked in red.

The last part of the research was intended to illustrate the way in which the beams cracked, according to the procedure described in Section 4.4. A picture of beam #2 is shown in Figure 35a, at the moment when the neutral axis of normal stress reaches the boundary of the OC and RPC layers, i.e., when $z_{c}=h_{\mathrm{RPC}}=70$ mm according to Figure 26. Similarly to beam #1 (Figure 25a), the correctness of Equation (10)—as used for the plotting the diagram in Figure 26—is also indirectly confirmed, because the cracks in the middle of beam #2 reach, at this moment, the areas just before the border of the OC and RPC layers, and the RPC layer behaves in a stable manner. The perpendicular cracks at the bottom of this beam had a maximum width of 2.3 mm at this moment. In turn, in Figure 35b at the end of testing beam #2, the two plastic hinges underneath the actuators can be clearly seen, as can the beginning of the RPC layer’s crushing. The bottom cracks already had a maximum width of 5.3 mm. This result is also consistent with what was stated for beam #1 (Figure 25b).

**Figure 36:**
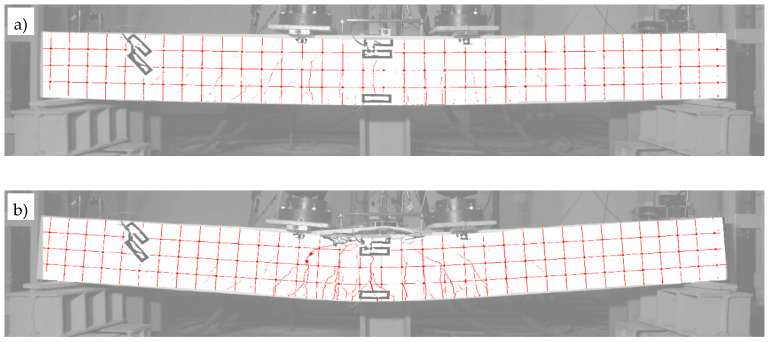
Views of the cracking of beam #3: (**a**) at the same deflection as for beam #2 in Figure 35a; (**b**) at the moment of the beam’s destruction. In order to increase the clarity of the figure, the instrumentation, the places where the strain gauges were glued, and the top concrete cover’s delimitation are painted in grey, and the positioning grid (10 cm mesh) and cracks are marked in red.

In turn, Figure 36 shows pictures of reference beam #3, which was made entirely of OC. For comparison purposes, in Figure 36a, beam #3 has the same deflection as beam #2 in Figure 35a, i.e., 21.6 mm. The maximum width of the perpendicular cracks at the bottom of beam #3 was approximately the same as at a comparable moment for beam #2, i.e., 2.2 mm, but the cracks reach a few centimetres lower than in beam #2. Figure 36b shows beam #3 exactly at the moment of the crushing of its compression zone and the exhaustion of its bending resistance. The maximum width of the cracks at the bottom was then 4.5 mm, i.e., about 15% less than at the end of the testing of beam #2.

Comparing the obtained results with the research from [9,19], which were discussed in more detail in Section 1 as being particularly thematically similar to the experiments presented in this article, the following facts can be emphasized. Contrary to our research, the maximum load for the three-point bent reinforced concrete slab elements from work [9], strengthened with the RPC layer from below (RE series), decreased from about 10% to 35%, depending on the RPC layer’s thickness when compared to the reference element made entirely of OC. Moreover, the reaching of their bending resistance was not clearly indicated by the cracks in the bottom view, as most of them initiated in the OC layer, and stopped their propagation at the layers’ boundary. On the other hand, as in the research presented in this paper, their ability to absorb energy in a post-critical state increased several times. In turn, according to the reference [19], which describes tests of a series of beams strengthened with the RPC layer both from the top and from the bottom, their maximum load in four-point bending increased, in the extreme case, to about 30%, compared to the reference beam made entirely of OC, except for only one beam with the thinnest 20 mm RPC layer from below. Similar to the research presented in [9] and in this article, the propagation of most of the cracks appearing in the OC layer of the beams also stopped at the border with the RPC, which confirms that the method of strengthening with RPC from below should be avoided for the safety reasons. On the other hand, the ratio of the measured maximum deflections in relation to the deflections at the beginning of the yielding of the tension steel rebars was lower according to [19] in all of the strengthened beams than in the reference beam. Thus, many differences can be noticed in the experimental results illustratively compared. According to the authors, one of the reasons for this may be the fact that, in [9], the applied reinforcement ratio was approximately 2.5%; in [19], it was approximately 0.45%; and in this article, it was approximately 1.7%. However, the last one was selected on the basis of a preliminary analysis of the optimal reinforcement ratio in terms of costs and bending resistance. Moreover, in this study, the dense system of stirrups (connecting both layers of the beams along their entire length) and the dispersed reinforcement in the RPC were used in order to increase the stability of the thin compressed RPC layer. As a result, an increase in the bending resistance of the reinforced elements was obtained, as well as an increase in the ability to absorb energy in a post-critical state compared to the reference element. In conclusion, it should be absolutely stated that the current state of knowledge, despite many interesting examples of experiments, indicates the need for more systematic research in this range; among others, in the context of the above-mentioned issues and in order to develop further the technical recommendations and design standards for this particular case of composite OC–RPC beams.

## 6. Conclusions

In the summary of the article, there are some preliminary conclusions resulting from the tests which were carried out which should be stressed. The most important of them are:

(1) The use of a relatively thin RPC layer of a compressive strength of 200 MPa in the compression zone of beams #1 and #2 contributed to a significant increase in their absorption of energy, and their very stable behaviour in the post-critical state compared to reference beam #3, in which only OC was used. For this purpose, the RPC layer also had to be protected against local debonding and buckling by a dense system of the stirrups penetrating the RPC and OC layers, and the dispersed steel fibre reinforcement in the RPC mix was used against local premature crushing. In such a case, the RPC layer placed in the compression zone of the beam allowed the structural capability of the high-ductility reinforcing steel to be fully utilised.
(2) In the design of the test beams with the RPC strengthening, it was decided to use a reinforcement ratio that was possibly close to the optimal one, taking into account the criteria of maintaining the bending resistance of beam and the cost of materials. The calculations gave a result for which the changes in the cost of the fabrication of the analysed beams with RPC of the desired bending resistance did not change significantly for a tension reinforcement ratio greater than 0.015.
(3) The original proposal for a visualization of the beams’ cracking by means of photogrammetry may allow us to analyse the processes of this type at any stage of loading laboratory tested concrete structural elements using available and relatively cheap photographic equipment. The applied research procedure proved to be particularly effective in assessing the development of the cracks in the beams in their post-critical state, where contact measurement is not possible for safety reasons, due to the high probability of the sudden exhaustion of the load resistance.
(4) The positions of the neutral axis of normal stress in the bent beams’ cross-sections were calculated based on the strains measured, showing that this axis is situated higher in the beams with the top RPC layer compared to the reference beam made entirely of OC. This difference has a noticeable impact on the lever arm of the stress-compressive and tensile-resultant forces, where it is grater in beams #1 and #2 than in beam #3. This translates into an approximately 12% increase in the global stiffness and bending resistance of the beams with the RPC.
(5) The comparative results of the ultrasonic wave velocity changes have indirectly confirmed the difference in the position of the normal stress neutral axis for the beams with and without the strengthening RPC layer. The ultrasonic measurements have also showed the perspective possibilities of using the relatively simple measurements of longitudinal wave velocity changes along the height of the beams in the diagnostics of this type of structural elements under bending, as well as in the indirect evaluation of the degree of the utilisation of their bending resistance.
(6) The construction solution proposed in the article may prove to be helpful in the design of new buildings where the height of the structural members’ cross-section is a limitation, or to ensure the stable behaviour of the structure in the post-critical state. This solution may also be useful for the strengthening of existing reinforced OC beams; however, this would require further research and analysis in relation to the appropriate bonding of the new RPC layer to the existing OC element.

## Figures and Tables

**Table 1:** Chemical composition of the RPC ingredients (percentage by weight).

Component	SiO_2_	Fe_2_O_3_	Al_2_O_3_	CaO	MgO	SO_3_	Na_2_O
Cement	21.83	2.00	4.38	65.68	0.93	3.29	0.29
Quartz powder	99.0	0.05	0.29	<0.1	<0.1	–	0.2
Quartz sand	98.6	0.03	0.75	–	–	–	–

**Table 2:** RPC mixture composition.

Component	Weight Fraction Related to the Mass of Cement
Cement	1.00
Silica fume	0.20
Quartz powder	0.12
Quartz sand	1.03
Water	0.24
Steel fibres	0.27
Superplasticiser	0.025

**Table 3:** Results of compressive and flexural strength tests of the RPC.

Age of Concrete [days]	Compressive Strength [MPa]	Flexural Strength [MPa]
1	95.2	36.0
2	145.7	48.2
7	166.8	49.6
28	197.9	51.9

**Table 4:** Total porosity and percentage of pores, depending on the diameter in the RPC after 28 days.

Total Porosity [%]	Percentage of Total Pore Volume Depending on the Diameter [%]				
	<20 nm	20–200 nm	200–2000 nm	2000–20,000 nm	>20,000 nm
4.4	77.1	8.0	3.6	2.5	8.8

**Table 5:** Total load, bending moment, deflection and curvature for the selected states of beam #1.

Description of the Beam’s State	$\mathrm{Total}\;\mathrm{Load}\;F_{\left(1\right)}+F_{\left(2\right)}\;[\mathrm{kN}]$	$\mathrm{Bending}\;\mathrm{Moment}\;\mathrm{at}\;\mathrm{the}\;\mathrm{Middle}\;\mathrm{of}\;\mathrm{Span}\;M_{\left(span\right)}\;[\mathrm{kNm}]$	$\mathrm{Deflection}\;\mathrm{at}\;\mathrm{the}\;\mathrm{Middle}\;\mathrm{of}\;\mathrm{Span}\;u_{\left(span\right)}^{*}\;[\mathrm{mm}]\;$	$\mathrm{Curvature}\;\mathrm{at}\;\mathrm{the}\;\mathrm{Middle}\;\mathrm{of}\;\mathrm{Span}\;\kappa_{\left(span\right)}$ $\;[{\mathrm{mm}}^{-1}]\times 10^{-5}$
Cracking moment	38	22.8	1.0	0.084
Steel yield point in the bottom reinforcing bars	394.1	236.5	17.6	1.524
Neutral axis reaches the boundary of the layers	389.1	233.5	24.8	3.162
End of the actuators’ travel range	422.3	253.4	278.5	ND

**Table 6:** Total load, bending moment, deflection, and curvature for the selected states of beams #2 and #3.

Description of the Beams’ State	$\mathrm{Total}\;\mathrm{Load}\;F_{\left(1\right)}+F_{\left(2\right)}\;[\mathrm{kN}]$		$\mathrm{Bending}\;\mathrm{Moment}\;\mathrm{at}\;\mathrm{the}\;\mathrm{Middle}\;\mathrm{of}\;\mathrm{Span}\;M_{\left(span\right)}\;[\mathrm{kNm}]$		$\mathrm{Deflection}\;\mathrm{at}\;\mathrm{the}\;\mathrm{Middle}\;\mathrm{of}\;\mathrm{Span}\;u_{\left(span\right)}^{*}\;[\mathrm{mm}]$		$\mathrm{Curvature}\;\mathrm{at}\;\mathrm{the}\;\mathrm{Middle}\;\mathrm{of}\;\mathrm{Span}\;\kappa_{\left(span\right)}$ $\;[{\mathrm{mm}}^{-1}]\times 10^{-5}$	
Beam	#2	#3	#2	#3	#2	#3	#2	#3
Cracking moment in stage 1	39.0	38.0	23.4	22.8	1.1	1.1	0.081	0.093
Maximum load in stage 1	52.2	50.7	31.3	30.4	1.6	1.7	0.132	0.153
Maximum load in stage 2	122.0	121.0	73.2	72.6	ND	4.6	ND	0.448
Maximum load in stage 3	191.8	191.5	115.1	114.9	6.2	7.6	0.508	0.737
Steel yield point in the bottom reinforcing bars in stage 4	397.1	372.5	238.3	223.5	15.6	18.3	1.371	1.687
Neutral axis reaches the boundary of the layers in beam #2 during stage 4	397.3	ND	238.4	ND	21.6	ND	3.042	ND
End of stage 4: end of the actuators’ travel range for beam #2 and destruction of beam #3.	419.6	361.6	251.8	217.0	178.5	71.5	ND	7.820

## Appendix A

### List of Symbols

$A_{s}$: the cross-sectional area of the tension rebars; [m^2^];
C: the objective function describing the cost of the beam’s unit section;
$C_{\mathrm{OC}}$: the price per m^3^ of OC;
$C_{\mathrm{RPC}}$: the price per m^3^ of RPC;
$C_{s}$: the price per m^3^ of reinforcing steel;
$E_{0}$: the dynamic modulus of the elasticity of the material in an undamaged (virgin) state; [Pa];
$E_{D}$: the dynamic modulus of the elasticity of the material in a damaged state; [Pa];
$E_{c\;\mathrm{RPC}}$: the modulus of the elasticity of RPC; [Pa];
$E_{s}$: the modulus of the elasticity of the reinforcing steel; [Pa];
$F_{\left(1\right)}$, $F_{\left(2\right)}$: the loads from the actuators; [N];
$F_{c}$: the resultant force of normal stress in the compressed concrete; [N];
$F_{s}$: the resultant force of normal stress in the tension steel rebars; [N];
M: the bending moment in the beam’s cross-section; [Nm];
$M_{R}$: the beam’s bending moment resistance at the limit state, according to Figure 4a; [Nm];
$M_{\left(\mathrm{span}\right)}$: the bending moment at the middle of beam’s span; [Nm];
a: the distance of center of gravity of longitudinal main rebars’ cross-section from the tension face of the beam; [m];
$a_{2}$: the distance of the center of gravity of the longitudinal secondary rebars’ cross-section from the compression face of the beam; [m];
b: the width of the beam’s cross-section; [m];
c: the dimensionless objective function proportional to C for the given values of $C_{\mathrm{RPC}}$, $C_{\mathrm{OC}}$, $C_{s}$, $f_{y}$, $h_{\mathrm{RPC}}$, d, a, and $M_{R}$; [-];
$c_{L0}$: the longitudinal wave velocity in the undamaged (virgin) material; [m/s];
$c_{\mathrm{LD}}$: the longitudinal wave velocity in the damaged material; [m/s];
d: the effective depth of the beam’s cross-section; [m];
$f_{c\;\mathrm{RPC}}$: the compressive strength of RPC; [Pa];
$f_{y}$: the yield point of the reinforcing steel; [Pa];
h: the height of the beam’s cross-section; [m];
$h_{\mathrm{RPC}}$: the thickness of the RPC layer; [m];
$u_{\left(1\right)}$, $u_{\left(2\right)}$: the deflection of the beam under the actuators; [m];
$u_{\left(\mathrm{span}\right)}$: the deflection of the beam at the middle of the span; [m];
$u_{\left(\mathrm{span}\right)}^{*}$: the corrected deflection of the beam at the middle of the span determined in relation to the actual support position; [m];
$u_{\left(\mathrm{support}\_1\right)}$, $u_{\left(\mathrm{support}\_2\right)}$: the deflection of the beam at the axis of the supports; [m];
x: the horizontal coordinate used in the photogrammetric analysis; [px];
$z^{*}$: the vertical coordinate used in the photogrammetric analysis; [px];
$z_{c}$: the position of the neutral axis as its distance from the beam’s compression face; [m];
$\varepsilon_{\left(1\right)}$, $\varepsilon_{\left(2\right)}$, $\varepsilon_{\left(3\right)}$, $\varepsilon_{\left(4\right)}$: the normal strains along the beam’s axis at the middle of the span on the upper surface of the beam (compression face); at the level of the longitudinal upper rebars; at the level of the 10.5 cm from the upper surface of the beam; and at the level of the longitudinal bottom rebars, respectively; [-];
$\varepsilon_{c\;\mathrm{LS}}$: the normal strain in the concrete at the compression face of the beam, at the moment of the reaching of the limit state, according to Figure 4a; [-];
$\varepsilon_{s}$: the normal strain in the tension rebars; [-];
$\varepsilon_{s\;\mathrm{LS}}$: the normal strain in the tension rebars at the moment of the reaching of the limit state, according to Figure 4a; [-];
$\varepsilon_{u}$: the normal strain in the rebars at the maximum load; [-];
$\kappa_{\left(\mathrm{span}\right)}$: the curvature of the beam’s upper surface at the middle of the span; [m^−1^];
ρ: the longitudinal tension reinforcement ratio; [-];
$\sigma_{c\;\mathrm{LS}\;}$: the stress in the concrete at the compression face of the beam at the moment of the reaching of the limit state, according to Figure 4a; [Pa];
$\sigma_{S}$: the normal stress in the tension rebars; [Pa].
